# Review of clinical trials and guidelines for children and youth with mucopolysaccharidosis: outcome selection and measurement

**DOI:** 10.1186/s13023-024-03364-x

**Published:** 2024-10-23

**Authors:** Alison H. Howie, Kylie Tingley, Michal Inbar-Feigenberg, John J. Mitchell, Kim Angel, Jenifer Gentle, Maureen Smith, Martin Offringa, Nancy J. Butcher, Philippe M. Campeau, Pranesh Chakraborty, Alicia Chan, Dean Fergusson, Eva Mamak, Peyton McClelland, Saadet Mercimek-Andrews, Aizeddin Mhanni, Zeinab Moazin, Cheryl Rockman-Greenberg, C. Anthony Rupar, Becky Skidmore, Sylvia Stockler, Kednapa Thavorn, Alexandra Wyatt, Beth K. Potter, INFORM RARE Network

**Affiliations:** 1 School of Epidemiology and Public Health, University of Ottawa, Ottawa, Canada; 2 Division of Clinical and Metabolic Genetics, Hospital for Sick Children, Toronto, Canada; 3 McGill University Health Centre, Montreal, Canada; 4 The Canadian MPS Society, Vancouver, Canada; 5 Patient Partner, Vancouver, Canada; 6 Patient Partner, Canadian Organization for Rare Disorders, Ottawa, Canada; 7 Child Health Evaluative Sciences, The Hospital for Sick Children Research Institute, Toronto, Canada; 8 Hospital for Sick Children, Toronto, Canada; 9 Université de Montréal, Montréal, Canada; 10 Children’s Hospital of Eastern Ontario, Ottawa, Canada; 11 Department of Medical Genetics, University of Alberta, Edmonton, Canada; 12 Ottawa Hospital Research Institute, Ottawa, Canada; 13 Department of Psychology, Hospital for Sick Children, Toronto, Canada; 14 Max Rady College of Medicine, Winnipeg, Canada; 15 Department of Pediatrics and Child Health, University of Manitoba, Winnipeg, Canada; 16 Department of Pathology and Laboratory Medicine, Western University, London, Canada; 17 Independent Information Specialist, Ottawa, Canada; 18 BC Children’s Hospital, Vancouver, Canada; 19 School of Epidemiology and Public Health, University of Ottawa, 600 Peter Morand Crescent, Ottawa, ON K1G 5Z3 Canada

**Keywords:** Mucopolysaccharidosis, Core outcome set, Outcomes

## Abstract

**Background:**

To inform the development of a core outcome set (COS) for children and youth with mucopolysaccharidoses (MPS), we aimed to identify all outcomes and associated outcome measurement instruments that are reported in recent clinical trials and recommended as measurements in clinical management guidelines.

**Methods:**

To identify English-language clinical trials and guidelines pertaining to MPS published between 2011 and mid-2021, we applied a comprehensive peer-reviewed search strategy to relevant databases and registers on May 16, 2021. Two reviewers independently screened retrieved citations and then full-text articles to determine eligibility for inclusion. From articles meeting inclusion criteria, we extracted details of the study design, population, intervention, and comparator, along with verbatim outcomes and associated outcome measurement instruments. Outcomes were organized into domains within five a priori core areas: life impact, pathophysiological manifestations, growth and development, resource use, and death. We conducted descriptive analyses at the study level, grouping articles arising from the same study.

**Results:**

From 2593 unique citations, 73 articles from 61 unique studies were included in the review, pertaining to all MPS subtypes except for exceptionally rare subtypes. Eighty-four unique outcomes were reported across the studies, 33 (39%) of which were reported by three or fewer studies. Most outcomes (55; 65%) were in the pathophysiological manifestations core area, followed by life impact (17; 20%) and growth and development (10; 12%); one outcome each pertained to resource use and death. The most frequently reported outcomes were general adverse events (45; 74%), immune-related adverse events (39; 64%), and urinary glycosaminoglycans (38; 62%). Substantial variability existed in the reporting of outcome measurement instruments. Some differences in outcome reporting were observed by MPS subtype and publication year.

**Discussion:**

Outcomes reported in clinical trials and guidelines for MPS in children and youth vary considerably and largely focus on pathophysiological manifestations. A COS is needed to standardize the selection and measurement of meaningful outcomes across future studies. We will present the outcomes identified in this review to knowledge users as part of a consensus process to select the most critical outcomes for inclusion in the COS.

*Trial Registration* The protocol for this study was registered in PROSPERO (CRD42021267531) and in the COMET Database.

**Supplementary Information:**

The online version contains supplementary material available at 10.1186/s13023-024-03364-x.

## Background

Mucopolysaccharidoses are a group of rare inherited lysosomal storage diseases characterized by an inability or impaired ability to break down complex sugars called glycosaminoglycans [1]. The resulting accumulation of glycosaminoglycans leads to progressive, multi-organ damage and, for some individuals, premature death [1–4]. There are eight known subtypes of MPS (with some subtypes having multiple clinical phenotypes): MPS I, MPS II, MPS IIIA-D, MPS IVA-B, MPS VI, MPS VII, MPS IX, and MPS X [5, 6]. More recently, researchers have described MPS-plus, a condition with similar multisystem manifestations and clinical presentation to MPS but with additional unique features, including rapidly evolving heart defects and hematopoietic disorders [7]. While the presentation and severity of the disease vary both within and between subtypes, some clinical manifestations, such as dysostosis multiplex, cardiac involvement, ocular manifestations, and organomegaly are typically observed across all subtypes [1, 8, 9]. Neurological and cognitive involvement are present in some subtypes of MPS; thus, MPS subtypes (or their sub-phenotypes) can be broadly categorized as neuronopathic (severe MPS I (Hurler/MPS IH), MPS II, MPS IIIA-D, MPS VII) or non-neuronopathic (attenuated MPS I (Hurler-Scheie, Scheie/MPS IA), MPS IV, MPS VI, MPS IX, MPS X) [10].

Disease-modifying molecular and enzyme replacement therapies exist to treat some subtypes of MPS [11] and additional therapies are currently being studied [12–14]. The blood–brain barrier constitutes a significant obstacle for current therapies to reach the central nervous system and treat neurological manifestations [15]. At present, clinically available therapies are not curative, and several unmet needs persist, for example, pain, sleep disturbances, and impaired mobility [16–18]. Patient-oriented research is needed to identify and evaluate interventions that address these needs.

Clinical research in the field of rare diseases poses unique challenges, for example, difficulties with timely recruitment leading to small sample sizes, heterogeneous study populations, and challenges in identifying relevant long term health outcomes due to a scarcity of natural history data [19, 20]. Thus, there is limited high-quality evidence available to inform clinical and policy decision-making for rare disease therapies [21]. To make the best use of limited evidence, it is critical that study results can be compared and synthesized across studies [22]. However, in health research in general, variability in outcome measurement and the persistence of outcome-reporting bias make cross-study comparisons difficult [23]. The Core Outcome Measures in Effectiveness Trials (COMET) Initiative, launched in 2010, aims to address this by promoting the standardized collection and reporting of outcomes through the development and implementation of core outcome sets (COSs) [23]. A COS is defined as an agreed-upon, minimum set of outcomes that should be measured in all clinical trials for a particular condition [23]. It is strongly recommended that end users, including individuals with lived experience of the condition under study, are involved in the development of a COS to ensure that their perspectives on the outcomes are included [23]; for a pediatric COS, this includes children, youth, and caregivers [24].

To date, COSs exist for a few rare diseases, including medium-chain acyl-CoA dehydrogenase deficiency, phenylketonuria, and osteogenesis imperfecta [25, 26]; a COS for head, neck, and respiratory disease in MPS II is currently being developed [27]. However, at present, no COS exists for pediatric MPSs in general. A key first step in the development of a COS is to identify existing knowledge, including the number and types of outcomes incorporated in studies for the condition [23]. The objectives of this study were: i) to identify all outcomes measured in recent clinical trials and recommended for measurement in recent guidelines for pediatric MPS; and ii) to identify outcome measurement instruments used or recommended to evaluate such outcomes.

## Methods

The protocol for the development of the COS was registered in PROSPERO (CRD42021267531) and the COMET Database (https://www.comet-initiative.org/Studies/Details/1924). The review was carried out following the methods provided in the published protocol [28] and reported according to the Preferred Reporting Items for Systematic Reviews and Meta-Analyses Statements (PRISMA, PRISMA-S; Additional file 1) [29, 30]. Post hoc modifications are described in the relevant sections below.

### Patient engagement

**Fig. 1:**
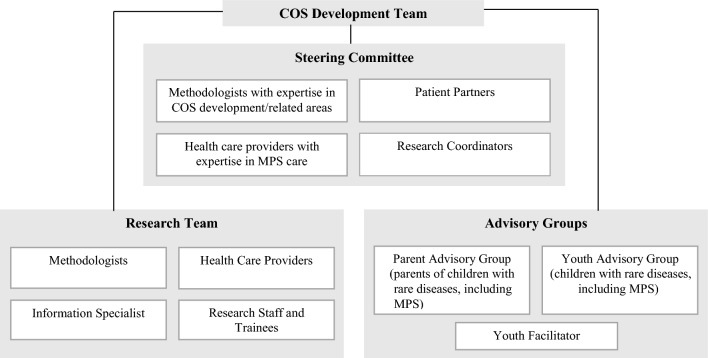
Core outcome set (COS) development team (adapted from Monga et al., [55])

The patient engagement strategy is described in the published protocol [28]. Briefly, patient partners, including patients, caregivers, and patient organization representatives, are investigators on the COS study and members of the COS Steering Committee (Fig. 1). These patient partners contributed to the conceptualization and decision-making for this review and are co-authors on this paper. Youth and parent advisors were involved in the later stages of the COS study, which relied on the review results, but did not directly contribute to the review itself.

### Eligibility criteria

Eligibility criteria for articles to include in this review were defined by PICOTS: population, intervention, comparator, outcomes, time frame, and study design [31].

- *Population:* Articles were originally considered eligible only if they were restricted to individuals aged 18 years or younger and diagnosed with MPS. However, when we initiated screening, we found the age criterion limiting, due to the rarity of MPS and the number of studies with mixed populations (i.e., children and adults combined). A post hoc amendment was therefore made to include clinical trials with mixed populations in which the majority of participants (> 50%) were 18 years or younger and outcomes were reported separately for children. Similarly, trial registry records with mixed populations that separately listed outcomes for pediatric patients were also considered eligible. Finally, guidelines that did not state that they were specific to children but that could apply to children were considered eligible, determined to be the case when any of the following were present: at least some of the recommendations included child-specific considerations; the recommended measurement frequency was post-diagnosis or on a regular basis; the assessments pertained to manifestations of MPS that are often experienced in the early stages of disease (e.g., hip dysplasia); or the assessments pertained to treatments that are recommended in the early stages of the disease.
- *Intervention and comparator:* Since the purpose of the review was to identify an exhaustive list of outcomes measured in recent studies of MPS, no restrictions were placed on eligible interventions or comparators.
- *Outcomes:* All articles with at least one outcome were considered eligible. A post hoc decision was made to exclude standard pharmacokinetic outcomes (e.g., half-life, area under the curve, volume of distribution) as these outcomes were not considered relevant for inclusion in a COS. Further, specific outcomes pertaining to anesthesia and peri- and intra-operative monitoring were considered beyond the scope of this work.
- *Time frame:* Aligned with our objective to identify outcomes from recent studies, articles published between January 1, 2011, and the May 2021 search dates (see “Information sources and search strategy**”**) were eligible for inclusion.
- *Study design:* We considered non-animal clinical trials and guidelines related to MPS to be eligible for inclusion. A guideline was defined as i) a clinical practice guideline from a professional association or from a governmental organization at a regional or higher level of geography that provides formal recommendations for clinical practice; ii) an article from a multi-disciplinary group of MPS experts making recommendations (broadly or for common manifestations) for the clinical management of MPS; or iii) an article from a multi-disciplinary group of MPS experts making recommendations about which outcomes to measure in research studies of MPS. To be eligible for inclusion, guidelines must have specified outcomes for monitoring patients and/or for evaluating the need for or effect of an intervention. Several guidelines presenting diagnostic recommendations were identified; thus, a post hoc modification was made to exclude guidelines in which the main focus was on the diagnosis, rather than the management of MPS.

Additional exclusion criteria were non-English articles (excluded due to lack of team expertise); abstracts or conference proceedings; studies of mixed populations of children and adults in which outcomes were not reported separately for children; and studies of multiple disorders in which outcomes were not reported separately for individuals with MPS. When a trial registry record corresponding to a published trial was identified, we only included the trial report. Lastly, guidelines that did not include information about how they were developed were not considered eligible.

### Information sources and search strategy

In consultation with the review team, an experienced information specialist (BS) drafted the search strategy. Following peer review by a second senior information specialist using the Peer Review of Electronic Search Strategies (PRESS) checklist, we incorporated suggested edits prior to executing the search [32].

We searched the following databases on the Ovid platform: Ovid MEDLINE® ALL, Embase Classic + Embase, and the Cochrane Central Register of Controlled Trials. In addition, we searched CINAHL using the Ebsco platform. We included both controlled vocabulary (e.g., “Mucopolysaccharidosis”) and free-text terms (e.g., “Hunter Syndrome”, “ARSB deficiency”) and applied study design filters (e.g., randomized and non-randomized controlled trials, clinical practice guidelines). We removed animal-only records and limited results to those published between 2011 and mid-2021. Searches were conducted on May 16, 2021. We used EndNote 9.3.3 (Clarivate) to deduplicate the search results and Covidence (Veritas Health Innovation) for screening. The full strategies are available in Additional file 2.

**Fig. 2:**
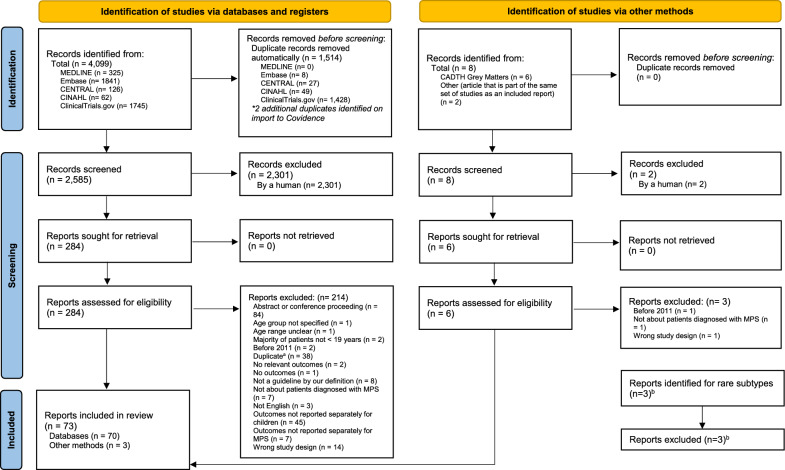
PRISMA 2020 flow diagram including searches of databases, registers and other sources. ^a^ Duplicates identified in phase 2 were either missed by de-duping tools or were clinical trial registration entries pertaining to published clinical trials included in the review. ^b^Discussions with clinician scientists regarding rare subtypes not already captured in the review revealed three potentially relevant reports. Since these reports did not meet eligibility criteria *and* did not include any new potentially relevant outcomes, they were not included in the review. *From:* Page MJ, McKenzie JE, Bossuyt PM, Boutron I, Hoffmann TC, Mulrow CD, et al. The PRISMA 2020 statement: an updated guideline for reporting systematic reviews. BMJ 2021; 372: n71. https://doi.org/10.1136/bmj.n71. For more information, visit: http://www.prisma-statement.org/

We performed a grey literature search to identify additional guidelines and trial registry records that were not captured in the aforementioned search. We searched relevant guideline registries listed in CADTH’s Grey Matters tool (e.g., Turning Research Into Practice (TRIP), ECRI Guidelines Trust) on May 22, 2021, and ClinicalTrials.gov on May 26, 2021. Due to the volume of ClinicalTrials.gov records, we included these entries in the main PRISMA flow diagram (Fig. 2).

In addition to the searches described above, we made a post hoc decision to ask clinician scientist members of the COS Steering Committee for any relevant articles pertaining to two exceptionally rare MPS subtypes (MPS IVB and MPS IX) that were not included in any of the eligible studies retrieved by the database and grey literature searches. We did not seek literature pertaining to MPS X and MPS-Plus given how recently they have been described in relation to the timing of our search. The purpose of this step was to identify any new outcomes that were not previously captured in the review and were potentially relevant for inclusion in the COS. We also made a post hoc decision not to consult members of the COS Steering Committee regarding guidelines (i.e., to review excluded guidelines and suggest additional guidelines) as several guidelines were identified in the searches and included in the review. Lastly, when grouping articles pertaining to the same study (see Sect. “Data synthesis”), we identified reports of related articles from the same overall study that were not picked up by the database search. When this occurred, if they met our inclusion criteria, they were included.

### Selection process

After citations were uploaded, Covidence software facilitated the automatic identification and removal of additional duplicate citations (beyond those identified in EndNote). We carried out a two-phase screening approach to identify eligible articles, with each phase involving screening by two reviewers working independently (among AHH, KT, PM, and AW). In phase one, we screened titles and abstracts (where available). Articles were moved to phase two if both reviewers agreed they were eligible. In instances of disagreement, a third reviewer was involved to reach a consensus. The full texts of potentially eligible articles were obtained. In phase two, we screened full-text articles following the same approach; we recorded reasons for exclusion in Phase 2 (see PRISMA diagram, Fig. 2). Study investigators were not contacted to answer queries related to eligibility. Phases one and two were piloted on a random sample of 20 and 10 articles, respectively. No modifications to the screening forms were required after the pilot testing.

### Data extraction

A single reviewer (among AHH, KT, and AW) extracted data from eligible articles in a Microsoft Excel form; a second team member (among AHH, KT, and ZM) verified all extracted information. We read articles in full and in instances of missing data, we consulted additional materials explicitly mentioned in the article where relevant (e.g., baseline characteristics of study participants previously described in another published report); authors were not contacted for further clarification. We piloted the data extraction form on a sample of 10 articles, including clinical trial reports, trial registry records, and guidelines. No major changes to the extraction form were made following pilot extractions.

The full data extraction form is available in Additional file 3. Briefly, data collection was centred around the PICOTS framework, with a detailed focus on outcomes. For clinical trials, we extracted all endpoints as reported by the authors. For guidelines, we extracted variables recommended for measurement or monitoring as part of the clinical management of MPS that are amenable to change or can develop over time. We extracted outcomes verbatim from each article, along with information on how the outcome was measured or recommended to be measured. Some guidelines did not formally recommend an outcome measurement instrument; in these cases, instruments that were provided as examples were included to obtain a comprehensive list. An outcome measurement instrument was defined as a questionnaire or specific functional test (e.g., six-minute walk test) and these were extracted where reported. We recorded whether the outcome measurement instrument was validated (as reported by the study authors), and any references provided. In some articles, authors reported a measurement instrument as an endpoint without explicitly indicating the outcome it measures, so that in our verbatim extraction, the same variable could be extracted as both an outcome and an outcome measurement instrument. For example, an echocardiogram was described in one article as among the “minimal parameters recommended for assessment” (i.e., as an outcome) [33] and in another article as an outcome measurement instrument to measure the outcome of cardiac function [34].

In addition, we extracted bibliographical study information, including title, year, and country of publication (determined by country of corresponding author) as well as information about the study population (i.e., MPS subtype, participant age).

### Data synthesis

For each article, the research team assigned a unique outcome label to each verbatim outcome that we had extracted, in consultation with members of the COS Steering Committee. This required grouping similar outcomes that were identified by different names but that measured the same overall concept. For example, “height”, “body height”, “standing height”, and “length” were grouped under the outcome label “height/length”. Further, we made decisions about the level of detail that was relevant in determining a unique outcome. For example, “gross motor skills”, “gait disturbance”, “ambulation”, and “balance” were grouped under the outcome label “mobility”. At this stage, we also reconciled outcome measurement instruments that were sometimes reported as outcomes (e.g., “echocardiogram” as an outcome was merged into the unique outcome, “cardiac function”). Finally, outcomes that were judged to be too broad (e.g., “symptoms”, “laboratory tests”) were removed at this stage.

We organized the resulting set of unique outcomes into the following core areas according to the Outcome Measures in Rheumatology (OMERACT) 2.0 filter adapted for pediatric studies: life impact, growth and development, pathophysiological manifestations, resource use, and death [35, 36]. Within each core area, outcomes were further categorized into domains [35]. We derived domains inductively for most core areas (i.e., based on the number and types of unique outcomes identified in our review), with the exception of pathophysiological manifestations, for which we used a published outcome taxonomy to group outcomes into domains for reporting [37]. Given that the vast majority of reported adverse events would be considered pathophysiological manifestations and considering the challenge in defining specific adverse events as part of the list of outcomes selected a priori for many clinical trials, we classified adverse events as part of the pathophysiological manifestations core area.

All data were analyzed descriptively in RStudio and Excel. Articles pertaining to the same study were grouped together for reporting. For example, a primary trial report, a report of a follow-up study from that trial, and a trial registry record for a second follow-up study would all be considered to be the same study. We reported the majority of the results by study but some descriptive statistics (e.g., publication year) were reported by article, for clarity. Outcomes were summarized overall and by MPS subtype and year of publication or registration. We made a post hoc decision not to summarize outcomes by participant age as this variable was not reported consistently across studies (i.e., studies reported age range, numbers of participants by age categories, mean age, or median age).

Because the purpose of the review was to identify all outcomes measured or recommended to be measured in the included studies, rather than to determine whether the studies’ findings about the interventions evaluated were based on sound methodology, we did not perform risk of bias assessments for clinical trials nor comprehensive appraisals of guidelines. However, recognizing that the outcomes recommended for measurement in guidelines may be influenced by the perspectives or interests of those involved in guideline development, we did include a post-hoc assessment of industry involvement in guidelines and included questions we adapted from the Editorial Independence domain of the Appraisal of Guidelines for REsearch & Evaluation II (AGREE-II) tool [38].

## Results

### Study selection

The full PRISMA flow diagram is presented in Fig. 2. Our search strategy returned 4107 citations of which 1514 were automatically removed as duplicates. Of the remaining 2593 unique citations screened for eligibility, 2303 were deemed ineligible based on titles and abstracts. We therefore screened 290 full-text articles, of which 73 met all eligibility criteria and were included in the review. We identified 61 unique studies among the 73 included articles.

Discussions with clinician scientists regarding rare subtypes (i.e., MPS IVB and MPS IX) revealed three potentially relevant articles for MPS IVB [39–41]. Because these articles did not meet the inclusion criteria and did not yield new outcomes, they were excluded from the analyses.

### Article and study characteristics

#### Articles

Descriptive characteristics of the included articles and studies are summarized in Table 1 (details for each article, Additional file 5, Table S5-1). Articles were most commonly clinical trial reports (36; 49%) and published in the United States (23; 43%).

**Table 1:** Descriptive characteristics of articles and unique studies included in the review

Characteristic	Frequency (%)^a^
*ARTICLES*	*(N* = *73)*
Study design	
Clinical trial Trial registry record Guideline	36 (49%) 19 (26%) 18 (25%)
Year of publication or registration	
2011–2014 2015–2018 2019–2021	22 (30%) 28 (38%) 23 (32%)
Country of publication^b,c^	
Argentina Brazil China France Japan Korea Poland South Africa Spain Sweden The Netherlands United Kingdom United States (Not applicable for trial registry records, n = 19)	1 (2%) 4 (7%) 1 (2%) 3 (6%) 3 (6%) 2 (4%) 1 (2%) 1 (2%) 2 (4%) 1 (2%) 4 (7%) 8 (15%) 23 (43%)
*UNIQUE STUDIES*	*(N* = *61)*
Type of intervention	
Enzyme replacement therapy Gene therapy Substrate reduction therapy Tumor necrosis factor alpha (TNF-α) inhibitor (Not applicable for guidelines, n = 18)	29 (67%) 8 (19%) 5 (12%) 1 (2%)
MPS subtype	
MPS (General)	3 (5%)
MPS I only	
MPS I-H MPS I-H, I-HS MPS I-H, I-HS, I-S MPS II only	5 (8%) 1 (2%) 1 (2%) 20 (33%)
MPS III only	
MPS IIIA	6 (10%)
MPS IIIB	3 (5%)
MPS IIIA-C	2 (3%)
MPS IIIA-D	2 (3%)
MPS IV only	
MPS IVA	7 (11%)
MPS VI only	5 (8%)
MPS VII only	2 (3%)
Multiple subtypes	
MPS I-H, II	1 (2%)
MPS I-H, I-HS, I-S, II, IVA, VI	1 (2%)
MPS I-H, I-HS, I-S, II, VI	1 (2%)
MPS II, III (unspecified)	1 (2%)
Age of trial participants^d^	
Children (< 19 years) only Children and adults (majority children) (Not applicable for trial registry records and guidelines, N = 32)	18 (62%) 11 (38%)
Sample size for completed trials (n = 29)^d,e^	
Median (Q1–Q3)	12 (7–25)
Industry involvement and editorial independence in guideline development^f^	
Direct industry funding	
Yes No	13 (72%) 5 (28%)
Author affiliation with industry	
Yes No	5 (28%) 13 (72%)
Statement of editorial independence	
Yes No	6 (33%) 12 (67%)
Competing interests recorded/discussed	
Recorded (none declared) Recorded Recorded and discussed	3 (17%) 12 (67%) 3 (17%)

*MPS* mucopolysaccharidosis

^a^Frequency and percentage unless otherwise specified. Percentages may not add to 100 due to rounding

^b^Not applicable to trial registry records. Percentages reported for those articles where this characteristic is applicable

^c^Determined by country of corresponding author

^d^Not applicable to trial registry records and guidelines

^e^Where there were multiple articles reporting on completed trials from the same study, only the sample size from the primary report was included

^f^Not appliable to clinical trials and trial registry records

#### Studies

The most common intervention investigated by the included studies was enzyme replacement therapy (29; 67%, Table 1). All but two MPS subtypes included in our search (MPS IVB and MPS IX) were represented in the review; the most studied subtype was MPS II (24 studies, including those focused only on MPS II and those focused on MPS II and other specific subtypes; 39%). The median (Q1 to Q3) sample size of all completed trials was 12 (7,25).

### Results of syntheses: outcomes and outcome measurement instruments

Across the 61 studies, 84 unique outcomes were reported (Table 2). Individual studies reported a median of 12 unique outcomes (range 1–40). No outcomes were reported by all studies. Thirty-three outcomes (39%) were reported by three or fewer studies.

**Table 2:** Frequency of outcome reporting across studies overall and with indication of which MPS subtypes are included

Outcome	# (%) of studies (n = 61)	MPS I	MPS II	MPS III	MPS IV	MPS VI	MPS VII	MPS General^a^
LIFE IMPACT (17 outcomes)	44 (72%)							
*Domain: Behaviour and emotional health*	*13 (21%)*							
Mood and behaviour changes	13 (21%)	✓	✓	✓				✓
*Domain: Child and caregiver/family support*	*40 (66%)*							
Activities of daily living	30 (49%)	✓	✓	✓	✓	✓	✓	✓
Quality of life	24 (39%)	✓	✓	✓	✓	✓		✓
Pain	13 (21%)	✓	✓		✓	✓		✓
Sleep apnoea/sleep disordered breathing	10 (16%)	✓	✓	✓	✓	✓		
Caregiver/family impact	6 (10%)		✓	✓				✓
Fatigue	3 (5%)				✓	✓	✓	
Sleep disturbances	2 (3%)			✓				
Autonomy/independence	1 (2%)		✓					
Overall health (parent assessed)	1 (2%)			✓				
Social impact and function	1 (2%)							✓
*Domain: Mobility and strength*	*24 (39%)*							
Endurance/exercise capacity	14 (23%)	✓	✓		✓	✓		
Mobility	14 (23%)	✓	✓	✓	✓	✓	✓	✓
Grip strength	5 (8%)	✓	✓		✓	✓		
Pinch strength	3 (5%)				✓	✓		
Strength	3 (5%)	✓			✓	✓		
Need for mobility aid	2 (3%)				✓	✓		
GROWTH AND DEVELOPMENT (10 outcomes)	47 (77%)							
*Domain: Physical growth and anthropometry*	*27 (44%)*							
Height/length	18 (30%)	✓	✓		✓	✓	✓	
Weight	16 (26%)	✓	✓	✓	✓	✓	✓	
Growth	15 (25%)	✓	✓	✓	✓	✓	✓	
Head circumference	10 (16%)		✓		✓	✓	✓	
Puberty	3 (5%)				✓	✓		
Arm span	2 (3%)	✓	✓					
*Domain: Cognition and development*	*36 (59%)*							
Cognitive function and early development	29 (48%)	✓	✓	✓	✓			✓
Fine-motor ability	5 (8%)				✓	✓	✓	✓
Speech abilities	5 (8%)		✓	✓	✓	✓		
School performance	1 (2%)				✓			
PATHOPHYSIOLOGICAL MANIFESTATIONS (55 outcomes)	60 (98%)							
*Domain: Adverse events/effects*	*51 (84%)*							
General adverse events^b^	45 (74%)	✓	✓	✓	✓	✓	✓	✓
Immune-related adverse events^b^	39 (64%)	✓	✓	✓	✓	✓	✓	
*Domain: Blood and lymphatic system outcomes*	*22 (36%)*							
General haematology	20 (33%)	✓	✓	✓	✓	✓	✓	
Biochemical evaluations	6 (10%)		✓	✓				
Hypoglycemia	1 (2%)	✓						
*Domain: Cardiac outcomes*	*32 (52%)*							
Cardiac function	32 (52%)	✓	✓	✓	✓	✓	✓	
*Domain: Congenital, familial and genetic outcomes*	*5 (8%)*							
Gene therapy monitoring outcomes	4 (7%)	✓		✓				
Successful donor chimerism	2 (3%)	✓						
*Domain: Ear and labyrinth outcomes*	*12 (20%)*							
Hearing	10 (16%)	✓	✓		✓	✓		
Ear, nose, and throat manifestations	5 (8%)		✓	✓	✓	✓		
*Domain: Endocrine outcomes*	*2 (3%)*							
Endocrine function	1 (2%)					✓		
Thyroid function	1 (2%)				✓			
*Domain: Eye outcomes*	*11 (18%)*							
Vision/eye health	11 (18%)	✓	✓		✓	✓	✓	✓
*Domain: Gastrointestinal outcomes*	*3 (5%)*							
Gastrointestinal manifestations	3 (5%)		✓	✓	✓			
*Domain: General outcomes*	*45 (74%)*							
Overall health (clinician assessment)	24 (39%)	✓	✓	✓	✓	✓	✓	✓
Organomegaly	24 (39%)	✓	✓	✓	✓	✓	✓	
Vital signs	23 (38%)	✓	✓	✓	✓	✓	✓	
Surgical intervention required^c^	9 (15%)	✓	✓		✓	✓		
Changes to concomitant medications	8 (13%)	✓	✓		✓	✓	✓	
Disease progression	7 (11%)	✓	✓	✓	✓	✓		✓
Oral health	5 (8%)		✓		✓	✓		
Facial features	4 (7%)		✓			✓		
Changes relative to personalized treatment goals	1 (2%)						✓	
Dentition	1 (2%)		✓					
Hair morphology	1 (2%)			✓				
Short neck	1 (2%)		✓					
*Domain: Hepatobiliary outcomes*	*4 (7%)*							
Liver function	4 (7%)	✓	✓			✓		
*Domain: Infection and infestation outcomes*	*3 (5%)*							
Infections (non-specific)	3 (5%)	✓	✓		✓	✓		
*Domain: Metabolism and nutrition outcomes*	*46 (75%)*							
Urinary glycosaminoglycans (GAGs)	38 (62%)	✓	✓	✓	✓	✓	✓	
Cerebrospinal fluid (CSF) GAGs	18 (30%)	✓	✓	✓				
Drug therapy monitoring (CSF/blood)	9 (15%)		✓	✓				
Blood GAGs	8 (13%)		✓	✓				
Enzyme activity	8 (13%)	✓	✓	✓				
*Domain: Musculoskeletal and connective tissue outcomes*	*29 (48%)*							
Upper limb joint function/range of motion (ROM)	13 (21%)	✓	✓		✓	✓	✓	
Lower limb joint function/ROM	12 (20%)	✓	✓		✓	✓		
Bone health^d^	10 (16%)	✓	✓		✓	✓		
Joint mobility/ROM (general)	10 (16%)	✓	✓		✓	✓		
Hernia	5 (8%)		✓		✓	✓		
Hip abnormalities	4 (7%)	✓			✓	✓		
Neuromuscular manifestations	4 (7%)		✓	✓	✓	✓		
Carpal tunnel syndrome	3 (5%)		✓			✓		
Hand joint mobility/ ROM	3 (5%)		✓			✓		
Valgus deformity of lower limbs	3 (5%)				✓	✓		
Arthropathy	2 (3%)	✓	✓					
*Domain: Nervous system outcomes*	*30 (49%)*							
General neurological manifestations	28 (46%)	✓	✓	✓	✓	✓		
Spinal manifestations	11 (18%)	✓	✓		✓	✓		
CSF pressure	2 (3%)	✓	✓					
Seizures (including due to epilepsy)	2 (3%)		✓	✓				
*Domain: Renal and urinary outcomes*	*17 (28%)*							
Urinalysis	14 (23%)	✓	✓	✓	✓		✓	
Renal function	3 (5%)		✓	✓		✓		
Urinary manifestations	1 (2%)		✓					
*Domain: Respiratory, thoracic and mediastinal outcomes*	*17 (28%)*							
Lung function	16 (26%)	✓	✓		✓	✓	✓	
Airway manifestations	6 (10%)		✓		✓	✓		
*Domain: Skin and subcutaneous tissue outcomes*	*1 (2%)*							
Skin manifestations	1 (2%)		✓					
*Domain: Vascular outcomes*	*1 (2%)*							
Hypertension	1 (2%)		✓					
HEALTH RESOURCE USE (1 outcome)	2 (3%)							
*Domain: Health resource use*	*2 (3%)*							
Health resource use	2 (3%)		✓			✓		
DEATH (1 outcome)	3 (5%)							
*Domain: Life expectancy*	*3 (5%)*							
Life expectancy	3 (5%)	✓		✓				

*CSF* cerebrospinal fluid; *GAGs* glycosaminoglycans; *MPS* mucopolysaccharidosis; *ROM* range of motion

^a^Some guidelines provided recommendations for MPS in general

^b^General adverse events were extracted as an outcome if they were reported in the methods or the results of a trial. Immune-related adverse events were only extracted if they were mentioned in the methods so as to not distort the findings based on the observed safety profile of the evaluated interventions

^c^Surgical intervention required captures both broad assessments (e.g., surgical procedures) and those related to specific manifestations (e.g., need for kyphosis surgery)

^d^Bone health captures both structural changes (e.g., cervical spine MRI, abnormal bone thickness and shape) and changes to bone composition (e.g., bone density, bone and cartilage metabolism)

#### Core areas

Outcomes were grouped into 25 domains within five core areas (Table 2). Most outcomes (55; 65%) were in the pathophysiological manifestations core area; 17 (20%) pertained to life impact; 10 (12%) growth and development; one (1%) resource use; and one (1%) death.

All but one study (60; 98%) reported an outcome within the pathophysiological manifestations core area. Within this core area, outcomes were most often reported in the following domains: adverse events/effects (51, including general and immune-related; 84% of all studies), metabolism and nutrition outcomes (46; 75%), and general outcomes (45; 74%). Most studies (47; 77%) also reported outcomes within the growth and development core area, with cognition and development being the most commonly reported domain (36; 59%). Nearly three-quarters of studies (44; 72%) reported outcomes pertaining to life impact, most of which were within the child and caregiver/family impact domain (40; 66%). Few studies reported outcomes pertaining to death (3; 5%) and resource use (2; 3%).

With respect to unique outcome labels, those reported by a majority (at least 31) of the 61 studies were general adverse events (74%), immune-related adverse events (64%), urinary glycosaminoglycans (62%), and cardiac function (52%).

#### Differences by MPS subtype

We observed some differences in outcomes reported across MPS subtypes (Table 2). For example, outcomes pertaining to mobility and strength and to musculoskeletal and connective tissues were reported less often for MPS III relative to MPS I, II, IV, and VI.

#### Changes in outcome reporting over time

The percentage of studies reporting outcomes in the growth and development core area increased steadily over time, with 59% of studies reporting such outcomes between 2011 and 2014, 83% between 2015 and 2018, and 90% between 2019 and 2021 (Table 3; further details by outcome, Additional file 5, Table S5-2). We observed no substantial differences over time in reporting of outcomes pertaining to life impact, pathophysiological outcomes, resource use, and death.

**Table 3:** Frequency of outcome reporting in five core areas, by year of publication

Core area	# (%) of studies (n = 61)^a^	# (%) of studies, 2011–2014 (n = 22)	# (%) of studies, 2015–2018 (n = 24)	# (%) of studies, 2019–2021 (n = 21)
Life impact	44 (72%)	16 (73%)	17 (71%)	16 (76%)
Growth and development	47 (77%)	13 (59%)	20 (83%)	19 (90%)
Pathophysiological manifestations	60 (98%)	22 (100%)	23 (96%)	21 (100%)
Resource use	2 (3%)	2 (9%)	0 (0%)	0 (0%)
Death	3 (5%)	1 (5%)	1 (4%)	1 (5%)

^a^Numbers of studies by publication years sum to more than 61 when added across the categories because some studies corresponded to more than one paper and the papers may have been published in different years

#### Outcome measurement instruments

We summarized outcome measurement instruments for all outcomes where at least one article reported or recommended measuring the outcome using a questionnaire or specific functional test (Additional file 5, Table S5-3). We identified 67 unique outcome measurement instruments or families of outcome measurement instruments (e.g., Pediatric Quality of Life Inventory (PedsQL)), of which seven were specific to MPS (e.g., Hunter Syndrome-Functional Outcomes for Clinical Understanding Scale, HS-FOCUS) [42]. Seven instruments were reported by authors as being validated (not specific to MPS). There was only one outcome, endurance/exercise capacity, that was reported by more than one study and for which all studies used the same outcome measurement instrument, the six-minute walk test (14 of 14 studies; 100%). However, several of these studies additionally measured this outcome using other instruments; thus, we identified seven unique outcome measurement instruments to measure endurance/exercise capacity. The outcomes with the most diverse set of outcome measurement instruments were cognitive function and early development (16 instruments across 29 studies), mood and behaviour changes (15 instruments across 13 studies), and activities of daily living (14 instruments across 30 studies). The most commonly used outcome measurement instruments for measuring cognitive function and early development were the Bayley Scales of Infant and Toddler Development [43] (17 of 29 studies; 59%) and the Kaufman Assessment Battery for Children [44] (13 of 29 studies; 45%).

## Discussion

To inform the development of a COS, we performed a comprehensive review of the literature to identify outcomes reported or recommended in recent clinical trials and guidelines pertaining to MPS in children and youth. We observed substantial heterogeneity in outcome reporting across studies. Of the 84 unique outcomes identified, over a third (39%) were reported by three or fewer studies, and no outcomes were consistently reported across all studies. When studies did report the same outcome, it was often inconsistently measured with a variety of measurement instruments. Given the multi-organ involvement and heterogeneity of MPS within and across subtypes, it is reasonable to expect that a variety of outcomes related to different bodily systems and aspects of health and functioning are measured. However, there is considerable diversity in the specific outcomes being reported within and across domains. For example, within the pathophysiological manifestations core area, we identified 11 unique outcomes pertaining to musculoskeletal and connective tissue outcomes. Despite an apparent agreement regarding the importance of musculoskeletal monitoring, the heterogeneity in specific outcomes being measured prevents the comparison and pooling of results across studies. This heterogeneity in outcome reporting has been reported in studies of a wide range of health conditions [45–47], including other rare diseases [26, 48]. These findings support the need for a COS to promote the standardization of outcome selection and measurement across future studies and thereby facilitate the comparison and synthesis of results.

Most outcomes identified in the review (65%) pertained to pathophysiological manifestations. All but one study (98%) reported an outcome within this core area; this finding was consistent over time. While many of the outcomes (e.g., pain, quality of life) could reasonably apply across a range of interventions, some outcomes (e.g., gene therapy monitoring, successful donor chimerism) were intervention specific. The dominance of outcomes related to pathophysiological manifestations is consistent with other reviews of studies across a range of pediatric conditions [36, 47, 48]. Given that all included trials investigated drug and gene therapies, it is reasonable to expect that a variety of pathophysiological parameters are measured to ascertain the efficacy and safety of the intervention. However, although we observed a slight increase in the percentage of studies reporting outcomes pertaining to life impact over the years, over 20% of studies published between 2019 and 2021 did not report even one such outcome. This suggests a potential lack of patient and caregiver engagement in MPS studies, particularly in the selection of outcomes, as research has shown that patients and caregivers often identify patient/caregiver-reported outcomes as most meaningful [49]. This underscores the need for a COS to incorporate the perspectives of key knowledge users, including those with lived experience of the condition and their caregivers, to ensure that meaningful patient-centred outcomes are further emphasized in future studies.

We noted some differences in outcome reporting across MPS subtypes. For example, outcomes related to mobility and strength and musculoskeletal and connective tissues were infrequently reported for MPS III, which we would expect given that progressive cognitive impairment is a dominant manifestation of MPS III rather than joint disease (although still present) [50–52]. However, as mentioned, these results must be interpreted with caution given the small number of included studies for rarer subtypes (e.g., MPS VII). For example, while no studies of MPS VII reported measuring cognitive function and early development, we should not conclude that this outcome is not considered relevant for this subtype as previous studies have indicated that neurologic involvement is often observed in patients with MPS VII [10]. In subsequent steps of the COS development process, it is critical to consider these differences and whether subtype-specific or subgroup-specific (e.g., neuronopathic and non-neuronopathic) recommendations are justified.

The results from the present review support findings from a recent review of outcomes related to head, neck, and respiratory disease in MPS II [27]. Despite the narrower focus of that review, the authors identified 41 unique outcomes across 18 studies, the majority of which (61%) pertained to pathophysiological manifestations [27]. Given its more specific focus and differences in time frame (period of 24 years) and study designs (more liberal inclusion of non-interventional studies), unsurprisingly, that review identified several outcomes in the pathophysiological manifestations core area, particularly from observational studies, that were not included in our review (e.g., bronchitis, rhinorrhea, and nasal polyps) [27]. Given our broader focus, we also assigned unique outcome labels differently than Metryka et al. with respect to the pathophysiological manifestations core area, for example, grouping outcomes pertaining to the ears, nose and under the single outcome label, “ear, nose, and throat manifestations”. However, there was substantial overlap in life impact outcomes between the two reviews, where, aligned with our findings, Metryka et al. also identified heterogeneity in outcome measurement instruments [27].

This review was conducted following rigorous methods as outlined in the published protocol [28] and recommended by COMET [23]. This included a comprehensive peer-reviewed search strategy, duplicate screening and data extraction, and multi-disciplinary engagement in decision-making about the grouping and synthesis of outcomes, guided by published frameworks [35, 37] and incorporating the views of clinician experts, patients, and caregivers on our Steering Committee. This review has some limitations. Our search strategy was restricted by time (past ten years) and study design (clinical trials and guidelines only), and we restricted screening to English-only records. While these criteria were implemented with the goal of identifying recent, potentially relevant outcomes for the COS, we acknowledge that we may have missed some outcomes, particularly for exceptionally rare subtypes (i.e., MPS IVB and MPS IX) that were not captured in the review. However, because of the significant clinical overlap between MPS IVA and MPS IVB, the literature reviewed on MPS IVA may also apply to MPS IVB [53]. This is an evolving field with limited literature about the rarest subtypes of MPS. For example, two subtypes have recently been described and were not included in our review: MPS X and MPS-Plus [6, 7]. As evidence becomes available regarding these subtypes, the applicability of the outcomes identified for other forms of MPS can be evaluated. Further, our restriction to recent publications, while by design, may have excluded outcomes included in studies specific to interventions (such as enzyme replacement therapy for some MPS subtypes) that were primarily evaluated prior to 2011. In addition, due to differences in the level of detail provided in outcome reporting across articles (i.e., some authors more clearly described outcomes and measurement tools than others), there was some subjectivity in how outcome details were extracted and further categorized by our team. However, outcome details were extracted in duplicate and outcome labels were assigned with a multi-disciplinary team of experts. Finally, while we aimed to identify recent outcomes, the search strategy was conducted in May 2021 and thus the most recently published articles are not included in the review. For example, guidelines for the clinical management of MPS III were published in 2022 [54]. However, the purpose of the review was to generate a list of previously measured outcomes to combine with outcomes derived from a complementary survey of patients and caregivers, in order to derive a long list of candidate outcomes to present in later steps of the COS development process (i.e., Delphi surveys). We recommend an update to the literature review when a full update to the COS is planned so that any new candidate outcomes can be considered for inclusion.

## Conclusions

Outcomes reported in studies of MPS in children and youth are highly variable and emphasize pathophysiological manifestations. Even when the same outcomes are reported across studies, the diversity in outcome measurement instruments used to measure these outcomes prevents synthesis and comparison. This study demonstrates that a COS is needed to standardize the selection and reporting of outcomes and measurement instruments across future studies in pediatric MPS. Our findings also highlight the need to understand the outcomes that are most meaningful to patients and caregivers and to ensure that patient-centred outcomes are emphasized in future studies. The results of this review will inform subsequent steps in the COS development process.

## Supplementary Information

Additional file 1.

Additional file 2.

Additional file 3.

Additional file 4.

Additional file 5.

## Data Availability

The review dataset is included in Additional file 4.

## Abbreviations

AGREE-II: Appraisal of guidelines for research and evaluation II
CIHR: Canadian Institutes of Health Research
COMET: Core outcome measures in effectiveness trials
COS: Core outcome set
MPS: Mucopolysaccharidosis
HS-FOCUS: Hunter syndrome-functional outcomes for clinical understanding scale
OMERACT: Outcome measures in rheumatology
PedsQL: Pediatric quality of life inventory
PICOTS: Population, intervention, comparator, outcomes, timeframe, study design
PRESS: Peer review of electronic search strategies
PRISMA: Preferred reporting items for systematic reviews and meta-analyses
TRIP: Turning research into practice
