# Supplementary material for: Review of clinical trials and guidelines for children and youth with mucopolysaccharidosis: outcome selection and measurement
Source: Orphanet J Rare Dis. 2024 Oct 23;19:393. doi: 10.1186/s13023-024-03364-x (PMC11520150; doi:10.1186/s13023-024-03364-x)
Supplement: Supplementary file 2 — Additional file 2. [file 13023_2024_3364_MOESM2_ESM.docx]

**ADDITIONAL FILE 2: SUPPLEMENTAL MATERIALS**

**Final Bibliographical Search Strategy (following peer review)**

INFORM – RARE – MPS

Final Strategy

2021 May 16

Ovid Multifile

Database: Embase Classic+Embase <1947 to 2021 May 14> , Ovid MEDLINE(R) ALL <1946 to May 14, 2021>, EBM Reviews - Cochrane Central Register of Controlled Trials <April 2021>

Search Strategy:

--------------------------------------------------------------------------------

1 exp Mucopolysaccharidosis/ (20992)

2 (mucopolysaccharidos#s or gargolylis* or gargoylis*).tw,kf. (13495)

3 ((alpha-l-iduronidase or iduronidase) adj2 (disease? or syndrome? or deficien*)).tw,kf. (294)

4 ((Hurler* or Hurler-Scheie* or Pfaundler-Hurler* or Scheie* or Schleie*) adj2 (disease? or syndrome?)).tw,kf. (2935)

5 (helmholtz harrington adj2 (disease? or syndrome?)).tw,kf. (0)

6 (chondroosteodysplas* or chondro-osteodysplas* or chondroosteodystroph* or chondro-osteodystroph* or chondroosteoplas* or chondro-osteoplas* or dysostos#s multiplex).tw,kf. (804)

7 (lipochondrodystroph* or lipo-chondrodystroph*).tw,kf. (435)

8 mckusick 25280.tw,kf. (16)

9 ((I2S or iduronate* or "iduronate 2" or sulfoiduronate* or sulfo-iduronate*) adj2 deficien*).tw,kf. (273)

10 (Hunter* adj2 (disease? or syndrome?)).tw,kf. (2546)

11 hunter* glossit#s.tw,kf. (23)

12 ((heparan sulfate or heparan sulphate or heparan sulfamidase or heparan sulphamidase or heparitin sulfate or heparitin sulphate or heparan-alpha-glucosaminide n-acetyltransferase) adj2 (deficien* or storage disease?)).tw,kf. (263)

13 heparitinuri*.tw,kf. (0)

14 mckusick 30990.tw,kf. (0)

15 ((sanfilippo* or san filippo*) adj2 deficien*).tw,kf. (27)

16 ((n-acetyl-alpha-d-glucosaminidase or n-acetylglucosamine-6-sulfat* or n-acetylglucosamine-6-sulphat* or NAGLU) adj2 deficien*).tw,kf. (105)

17 ((polydystrophic* or poly-dystrophic*) adj2 (dwarf* or oligophreni* or oligo-phreni*)).tw,kf. (37)

18 ((acetyl-CoA alpha-glucosaminide n-acetyltransferase or heparan-alpha-glucosaminide n-acetyltransferase or HGSNAT) adj2 deficien*).tw,kf. (25)

19 ((n-acetylglucosamine-6-sulfate sulfatase or n-acetylglucosamine-6-sulphate sulphatase or glucosamine n-acetyl-6-sulfatase or glucosamine n-acetyl-6-sulphatase or GNS) adj2 deficien*).tw,kf. (22)

20 (morquio* adj2 (disease? or syndrome?)).tw,kf. (1978)

21 ((galactosamine-6-sulfatase or galactosamine-6-sulphatase or n-acetylgalactosamine-6-sulfate sulfatase or n-acetylgalactosamine-6-sulphate sulphatase or GALNS) adj2 deficien*).tw,kf. (177)

22 (kerato sulfatur* or kerato sulphatur* or keratosulfatur* or keratosulphatur*).tw,kf. (10)

23 ((osteochondrodystroph* or osteo-chondrodystroph*) adj1 deformans).tw,kf. (15)

24 familial osseous dystroph*.tw,kf. (2)

25 (eccentro?steochondrodysplas* or eccentro-osteochondrodysplas* or eccentro-osteo-chondrodysplasia* or eccentroosteo-chondrodysplas*).tw,kf. (136)

26 (maroteaux lamy adj2 (disease? or syndrome?)).tw,kf. (723)

27 ((arylsulfatase b or arylsulphatase b or n-acetylgalactosamine-4-sulfat* or n-acetylgalactosamine-4-sulphat* or ARSB) adj2 deficien*).tw,kf. (288)

28 mckusick 25320.tw,kf. (0)

29 (sly adj2 (disease? or syndrome?)).tw,kf. (228)

30 ((beta-glucuronidase or GUSB) adj2 deficien*).tw,kf. (457)

31 (scleromyxedem* or sclero-myxedem*).tw,kf. (925)

32 (lichen adj1 (fibromucinoidos* or fibro-mucinoidos* or myxedematos* or myxoedematos* or myxooedematos* or myxo-oedematos*)).tw,kf. (452)

33 ((mucinosis or mucinous*) adj3 (papular* or papulos*)).tw,kf. (453)

34 ((myxedema* or myxoedema* or myxooedema* or myxo-oedema*) adj3 (papular or papulos*)).tw,kf. (95)

35 (MPS 1? or MPS1? or MPS I? or MPSI? or "MPS1-H" or "MPSIH/S" or "MPS1H/S" or "MPS1-HS" or "MPS1-S" or MPSIS or MPS 2 or MPS2 or MPS II or MPSII or MPS 3? or MPS3? or MPS III? or MPSIII? or MPS 4? or MPS4? or MPS IV? or MPSIV? or MPS 6 or MPS6 or MPS VI or MPSVI or MPS 7 or MPS7 or MPS VII or MPSVII or MPS 9 or MPS9 or MPS IX or MPSIX).tw,kf. (15766)

36 or/1-35 [MPS] (34448)

37 exp Adult/ not (Adolescent/ or exp Child/ or exp Infant/) (14173618)

38 36 not 37 [ADULT-ONLY REMOVED] (29246)

39 exp Animals/ not Humans/ (16884742)

40 38 not 39 [ANIMAL-ONLY REMOVED] (22104)

41 limit 40 to yr="2011-current" (8810)

42 (controlled clinical trial or randomized controlled trial or pragmatic clinical trial or equivalence trial).pt. (1225827)

43 "Clinical Trials as Topic"/ (311671)

44 exp "Controlled Clinical Trials as Topic"/ (371070)

45 (randomi#ed or randomi#ation? or randomly or RCT or placebo*).tw,kf. (3651523)

46 ((singl* or doubl* or trebl* or tripl*) adj (mask* or blind* or dumm*)).tw,kf. (725913)

47 trial.ti. (924003)

48 or/42-47 [RCT FILTER] (4600148)

49 41 and 48 [RCTs] (494)

50 controlled clinical trial.pt. (186233)

51 Controlled Clinical Trial/ or Controlled Clinical Trials as Topic/ (573312)

52 (control* adj2 trial).tw,kf. (674038)

53 Non-Randomized Controlled Trials as Topic/ (12538)

54 (nonrandom* or non-random* or quasi-random* or quasi-experiment*).tw,kf. (154090)

55 (nRCT or non-RCT).tw,kf. (987)

56 Controlled Before-After Studies/ (226950)

57 (control* adj3 ("before and after" or "before after")).tw,kf. (804059)

58 Interrupted Time Series Analysis/ (219841)

59 time series.tw,kf. (75339)

60 (pre- adj5 post-).tw,kf. (329864)

61 (pretest adj5 posttest).tw,kf. (17114)

62 Historically Controlled Study/ (237400)

63 (control* adj2 study).tw,kf. (547559)

64 Control Groups/ (124519)

65 (control* adj2 group?).tw,kf. (1555496)

66 trial.ti. (924003)

67 or/50-66 [nRCT FILTER] (4348515)

68 41 and 67 [nRCTs] (417)

69 exp Clinical Pathways/ (16157)

70 exp Clinical Protocols/ (298896)

71 Consensus/ (93400)

72 exp Consensus Development Conference/ (36824)

73 exp Consensus Development Conferences as Topic/ (27457)

74 exp Guideline/ (35712)

75 Guidelines as Topic/ (457489)

76 Practice Guidelines as Topic/ (474951)

77 Health Planning Guidelines/ (105477)

78 (Guideline or Practice Guideline or Consensus Development Conference or Consensus Development Conference, NIH).pt. (45370)

79 (position statement* or policy statement* or practice parameter* or best practice*).tw,kf. (92705)

80 (standards or guideline or guidelines).ti,kf. (258958)

81 ((practice or treatment* or clinical) adj guideline*).ab. (116036)

82 (CPG or CPGs).ti. (13249)

83 consensus*.ti,kf. (61894)

84 consensus*.ab. /freq=2 (65764)

85 ((critical or clinical or practice) adj2 (path or paths or pathway or pathways or protocol*)).tw,kf. (59083)

86 recommendat*.ti,kf. (100357)

87 (overview? adj2 guideline?).tw,kf. (308)

88 or/69-87 [CPG FILTER] (1538391)

89 41 and 88 [CPGs] (227)

90 49 or 68 or 89 [ALL STUDY DESIGNS] (878)

91 90 use medall [MEDLINE RECORDS] (332)

92 exp mucopolysaccharidosis/ (20992)

93 (mucopolysaccharidos#s or gargolylis* or gargoylis*).tw,kw. (13693)

94 ((alpha-l-iduronidase or iduronidase) adj2 (disease? or syndrome? or deficien*)).tw,kw. (297)

95 ((Hurler* or Hurler-Scheie* or Pfaundler-Hurler* or Scheie* or Schleie*) adj2 (disease? or syndrome?)).tw,kw. (2989)

96 (helmholtz harrington adj2 (disease? or syndrome?)).tw,kw. (0)

97 (chondroosteodysplas* or chondro-osteodysplas* or chondroosteodystroph* or chondro-osteodystroph* or chondroosteoplas* or chondro-osteoplas* or dysostos#s multiplex).tw,kw. (838)

98 (lipochondrodystroph* or lipo-chondrodystroph*).tw,kw. (490)

99 mckusick 25280.tw,kw. (16)

100 ((I2S or iduronate* or "iduronate 2" or sulfoiduronate* or sulfo-iduronate*) adj2 deficien*).tw,kw. (273)

101 (Hunter* adj2 (disease? or syndrome?)).tw,kw. (2592)

102 hunter* glossit#s.tw,kw. (24)

103 ((heparan sulfate or heparan sulphate or heparan sulfamidase or heparan sulphamidase or heparitin sulfate or heparitin sulphate or heparan-alpha-glucosaminide n-acetyltransferase) adj2 (deficien* or storage disease?)).tw,kw. (264)

104 heparitinuri*.tw,kw. (0)

105 mckusick 30990.tw,kw. (0)

106 ((sanfilippo* or san filippo*) adj2 deficien*).tw,kw. (27)

107 ((n-acetyl-alpha-d-glucosaminidase or n-acetylglucosamine-6-sulfat* or n-acetylglucosamine-6-sulphat* or NAGLU) adj2 deficien*).tw,kw. (105)

108 ((polydystrophic* or poly-dystrophic*) adj2 (dwarf* or oligophreni* or oligo-phreni*)).tw,kw. (37)

109 ((acetyl-CoA alpha-glucosaminide n-acetyltransferase or heparan-alpha-glucosaminide n-acetyltransferase or HGSNAT) adj2 deficien*).tw,kw. (25)

110 ((n-acetylglucosamine-6-sulfate sulfatase or n-acetylglucosamine-6-sulphate sulphatase or glucosamine n-acetyl-6-sulfatase or glucosamine n-acetyl-6-sulphatase or GNS) adj2 deficien*).tw,kw. (22)

111 (morquio* adj2 (disease? or syndrome?)).tw,kw. (1994)

112 ((galactosamine-6-sulfatase or galactosamine-6-sulphatase or n-acetylgalactosamine-6-sulfate sulfatase or n-acetylgalactosamine-6-sulphate sulphatase or GALNS) adj2 deficien*).tw,kw. (178)

113 (kerato sulfatur* or kerato sulphatur* or keratosulfatur* or keratosulphatur*).tw,kw. (10)

114 ((osteochondrodystroph* or osteo-chondrodystroph*) adj1 deformans).tw,kw. (15)

115 familial osseous dystroph*.tw,kw. (2)

116 (eccentro?steochondrodysplas* or eccentro-osteochondrodysplas* or eccentro-osteo-chondrodysplasia* or eccentroosteo-chondrodysplas*).tw,kw. (158)

117 (maroteaux lamy adj2 (disease? or syndrome?)).tw,kw. (748)

118 ((arylsulfatase b or arylsulphatase b or n-acetylgalactosamine-4-sulfat* or n-acetylgalactosamine-4-sulphat* or ARSB) adj2 deficien*).tw,kw. (290)

119 mckusick 25320.tw,kw. (0)

120 (sly adj2 (disease? or syndrome?)).tw,kw. (244)

121 ((beta-glucuronidase or GUSB) adj2 deficien*).tw,kw. (463)

122 (scleromyxedem* or sclero-myxedem*).tw,kw. (941)

123 (lichen adj1 (fibromucinoidos* or fibro-mucinoidos* or myxedematos* or myxoedematos* or myxooedematos* or myxo-oedematos*)).tw,kw. (472)

124 ((mucinosis or mucinous*) adj3 (papular* or papulos*)).tw,kw. (468)

125 ((myxedema* or myxoedema* or myxooedema* or myxo-oedema*) adj3 (papular or papulos*)).tw,kw. (96)

126 (MPS 1? or MPS1? or MPS I? or MPSI? or "MPS1-H" or "MPSIH/S" or "MPS1H/S" or "MPS1-HS" or "MPS1-S" or MPSIS or MPS 2 or MPS2 or MPS II or MPSII or MPS 3? or MPS3? or MPS III? or MPSIII? or MPS 4? or MPS4? or MPS IV? or MPSIV? or MPS 6 or MPS6 or MPS VI or MPSVI or MPS 7 or MPS7 or MPS VII or MPSVII or MPS 9 or MPS9 or MPS IX or MPSIX).tw,kw. (15836)

127 or/92-126 [MPS] (34537)

128 exp adult/ not exp juvenile/ (8052810)

129 127 not 128 [ADULT-ONLY REMOVED] (31013)

130 exp animal/ or exp animal experimentation/ or exp animal model/ or exp animal experiment/ or nonhuman/ or exp vertebrate/ (55743169)

131 exp human/ or exp human experimentation/ or exp human experiment/ (43449265)

132 130 not 131 (12295760)

133 129 not 132 [ANIMAL-ONLY REMOVED] (27567)

134 limit 133 to yr="2011-current" (12712)

135 exp randomized controlled trial/ or controlled clinical trial/ (1467044)

136 clinical trial/ (1551744)

137 exp "controlled clinical trial (topic)"/ (210467)

138 (randomi#ed or randomi#ation? or randomly or RCT or placebo*).tw,kw. (3714210)

139 ((singl* or doubl* or trebl* or tripl*) adj (mask* or blind* or dumm*)).tw,kw. (754692)

140 trial.ti. (924003)

141 or/135-140 [RCT FILTER] (5192747)

142 134 and 141 [RCTs] (861)

143 controlled clinical trial/ (557793)

144 "controlled clinical trial (topic)"/ (11575)

145 (control* adj2 trial).tw,kw. (1026477)

146 (nonrandom* or non-random* or quasi-random* or quasi-experiment*).tw,kw. (155103)

147 (nRCT or non-RCT).tw,kw. (989)

148 (control* adj3 ("before and after" or "before after")).tw,kw. (804063)

149 time series analysis/ (29066)

150 time series.tw,kw. (76343)

151 pretest posttest control group design/ (561)

152 (pre- adj5 post-).tw,kw. (329907)

153 (pretest adj5 posttest).tw,kw. (20804)

154 controlled study/ (8138197)

155 (control* adj2 study).tw,kw. (957555)

156 control group/ (124418)

157 (control* adj2 group?).tw,kw. (1556694)

158 trial.ti. (924003)

159 or/143-158 [nRCT FILTER] (11191228)

160 134 and 159 [nRCTs] (2037)

161 exp practice guideline/ (623500)

162 (position statement* or policy statement* or practice parameter* or best practice*).tw,kw. (93271)

163 (standards or guideline or guidelines).ti,kw. (287621)

164 ((practice or treatment* or clinical) adj guideline*).ab. (116036)

165 (CPG or CPGs).ti. (13249)

166 consensus/ (93400)

167 consensus*.ti,kw. (68924)

168 consensus*.ab. /freq=2 (65764)

169 ((critical or clinical or practice) adj2 (path or paths or pathway or pathways or protocol*)).tw,kw. (65909)

170 recommendat*.ti,kw. (103226)

171 (overview? adj2 guideline?).tw,kw. (312)

172 or/161-171 [CPG FILTER] (1212464)

173 134 and 172 [CPGs] (307)

174 142 or 160 or 173 [ALL STUDY DESIGNS] (2684)

175 174 use emczd [EMBASE RECORDS] (2059)

176 exp Mucopolysaccharidosis/ (20992)

177 (mucopolysaccharidos#s or gargolylis* or gargoylis*).ti,ab,kw. (13693)

178 ((alpha-l-iduronidase or iduronidase) adj2 (disease? or syndrome? or deficien*)).ti,ab,kw. (297)

179 ((Hurler* or Hurler-Scheie* or Pfaundler-Hurler* or Scheie* or Schleie*) adj2 (disease? or syndrome?)).ti,ab,kw. (2989)

180 (helmholtz harrington adj2 (disease? or syndrome?)).ti,ab,kw. (0)

181 (chondroosteodysplas* or chondro-osteodysplas* or chondroosteodystroph* or chondro-osteodystroph* or chondroosteoplas* or chondro-osteoplas* or dysostos#s multiplex).ti,ab,kw. (838)

182 (lipochondrodystroph* or lipo-chondrodystroph*).ti,ab,kw. (490)

183 mckusick 25280.ti,ab,kw. (16)

184 ((I2S or iduronate* or "iduronate 2" or sulfoiduronate* or sulfo-iduronate*) adj2 deficien*).ti,ab,kw. (273)

185 (Hunter* adj2 (disease? or syndrome?)).ti,ab,kw. (2592)

186 hunter* glossit#s.ti,ab,kw. (24)

187 ((heparan sulfate or heparan sulphate or heparan sulfamidase or heparan sulphamidase or heparitin sulfate or heparitin sulphate or heparan-alpha-glucosaminide n-acetyltransferase) adj2 (deficien* or storage disease?)).ti,ab,kw. (264)

188 heparitinuri*.ti,ab,kw. (0)

189 mckusick 30990.ti,ab,kw. (0)

190 ((sanfilippo* or san filippo*) adj2 deficien*).ti,ab,kw. (27)

191 ((n-acetyl-alpha-d-glucosaminidase or n-acetylglucosamine-6-sulfat* or n-acetylglucosamine-6-sulphat* or NAGLU) adj2 deficien*).ti,ab,kw. (105)

192 ((polydystrophic* or poly-dystrophic*) adj2 (dwarf* or oligophreni* or oligo-phreni*)).ti,ab,kw. (37)

193 ((acetyl-CoA alpha-glucosaminide n-acetyltransferase or heparan-alpha-glucosaminide n-acetyltransferase or HGSNAT) adj2 deficien*).ti,ab,kw. (25)

194 ((n-acetylglucosamine-6-sulfate sulfatase or n-acetylglucosamine-6-sulphate sulphatase or glucosamine n-acetyl-6-sulfatase or glucosamine n-acetyl-6-sulphatase or GNS) adj2 deficien*).ti,ab,kw. (22)

195 (morquio* adj2 (disease? or syndrome?)).ti,ab,kw. (1994)

196 ((galactosamine-6-sulfatase or galactosamine-6-sulphatase or n-acetylgalactosamine-6-sulfate sulfatase or n-acetylgalactosamine-6-sulphate sulphatase or GALNS) adj2 deficien*).ti,ab,kw. (178)

197 (kerato sulfatur* or kerato sulphatur* or keratosulfatur* or keratosulphatur*).ti,ab,kw. (10)

198 ((osteochondrodystroph* or osteo-chondrodystroph*) adj1 deformans).ti,ab,kw. (15)

199 familial osseous dystroph*.ti,ab,kw. (2)

200 (eccentro?steochondrodysplas* or eccentro-osteochondrodysplas* or eccentro-osteo-chondrodysplasia* or eccentroosteo-chondrodysplas*).ti,ab,kw. (158)

201 (maroteaux lamy adj2 (disease? or syndrome?)).ti,ab,kw. (748)

202 ((arylsulfatase b or arylsulphatase b or n-acetylgalactosamine-4-sulfat* or n-acetylgalactosamine-4-sulphat* or ARSB) adj2 deficien*).ti,ab,kw. (290)

203 mckusick 25320.ti,ab,kw. (0)

204 (sly adj2 (disease? or syndrome?)).ti,ab,kw. (244)

205 ((beta-glucuronidase or GUSB) adj2 deficien*).ti,ab,kw. (463)

206 (scleromyxedem* or sclero-myxedem*).ti,ab,kw. (941)

207 (lichen adj1 (fibromucinoidos* or fibro-mucinoidos* or myxedematos* or myxoedematos* or myxooedematos* or myxo-oedematos*)).ti,ab,kw. (472)

208 ((mucinosis or mucinous*) adj3 (papular* or papulos*)).ti,ab,kw. (468)

209 ((myxedema* or myxoedema* or myxooedema* or myxo-oedema*) adj3 (papular or papulos*)).ti,ab,kw. (96)

210 (MPS 1? or MPS1? or MPS I? or MPSI? or "MPS1-H" or "MPSIH/S" or "MPS1H/S" or "MPS1-HS" or "MPS1-S" or MPSIS or MPS 2 or MPS2 or MPS II or MPSII or MPS 3? or MPS3? or MPS III? or MPSIII? or MPS 4? or MPS4? or MPS IV? or MPSIV? or MPS 6 or MPS6 or MPS VI or MPSVI or MPS 7 or MPS7 or MPS VII or MPSVII or MPS 9 or MPS9 or MPS IX or MPSIX).ti,ab,kw. (15834)

211 or/176-210 [MPS] (34535)

212 limit 211 to yr="2011-current" (16716)

213 212 use cctr [CENTRAL RECORDS] (230)

214 91 or 175 or 213 [ALL DATABASES] (2621)

215 remove duplicates from 214 (2292) [TOTAL UNIQUE RECORDS]

216 215 use medall [MEDLINE UNIQUE RECORDS] (325)

217 215 use emczd [EMBASE UNIQUE RECORDS] (1841)

218 215 use cctr [CENTRAL UNIQUE RECORDS] (126)

***************************

CINAHL

| # | Query | Limiters/Expanders | Last Run Via | Results |
| --- | --- | --- | --- | --- |
| S78 | S46 OR S64 OR S77 | Expanders - Apply related words; Apply equivalent subjects  Search modes - Boolean/Phrase | Interface - EBSCOhost Research Databases  Search Screen - Advanced Search  Database - CINAHL | 62 |
| S77 | S40 AND S76 | Expanders - Apply related words; Apply equivalent subjects  Search modes - Boolean/Phrase | Interface - EBSCOhost Research Databases  Search Screen - Advanced Search  Database - CINAHL | 24 |
| S76 | S65 OR S66 OR S67 OR S68 OR S69 OR S70 OR S71 OR S72 OR S73 OR S74 OR S75 | Expanders - Apply related words; Apply equivalent subjects  Search modes - Boolean/Phrase | Interface - EBSCOhost Research Databases  Search Screen - Advanced Search  Database - CINAHL | 185,419 |
| S75 | TI overview# N2 guideline# OR AB overview# N2 guideline# | Expanders - Apply related words; Apply equivalent subjects  Search modes - Boolean/Phrase | Interface - EBSCOhost Research Databases  Search Screen - Advanced Search  Database - CINAHL | 171 |
| S74 | TI ( (critical or clinical or practice) N2 (path or paths or pathway or pathways or protocol*) ) OR AB ( (critical or clinical or practice) N2 (path or paths or pathway or pathways or protocol*) ) | Expanders - Apply related words; Apply equivalent subjects  Search modes - Boolean/Phrase | Interface - EBSCOhost Research Databases  Search Screen - Advanced Search  Database - CINAHL | 9,397 |
| S73 | TI recommendat* | Expanders - Apply related words; Apply equivalent subjects  Search modes - Boolean/Phrase | Interface - EBSCOhost Research Databases  Search Screen - Advanced Search  Database - CINAHL | 19,307 |
| S72 | TI consensus | Expanders - Apply related words; Apply equivalent subjects  Search modes - Boolean/Phrase | Interface - EBSCOhost Research Databases  Search Screen - Advanced Search  Database - CINAHL | 9,113 |
| S71 | TI CPG or CPGs | Expanders - Apply related words; Apply equivalent subjects  Search modes - Boolean/Phrase | Interface - EBSCOhost Research Databases  Search Screen - Advanced Search  Database - CINAHL | 358 |
| S70 | TI ( (practice or treatment* or clinical) W0 guideline* ) OR AB ( (practice or treatment* or clinical) W0 guideline* ) | Expanders - Apply related words; Apply equivalent subjects  Search modes - Boolean/Phrase | Interface - EBSCOhost Research Databases  Search Screen - Advanced Search  Database - CINAHL | 25,762 |
| S69 | TI standards or guideline or guidelines | Expanders - Apply related words; Apply equivalent subjects  Search modes - Boolean/Phrase | Interface - EBSCOhost Research Databases  Search Screen - Advanced Search  Database - CINAHL | 57,644 |
| S68 | TI ( (position W0 statement*) or (policy W0 statement*) or (practice W0 parameter*) or (best W0 practice*) ) OR AB ( (position W0 statement*) or (policy W0 statement*) or (practice W0 parameter*) or (best W0 practice*) ) | Expanders - Apply related words; Apply equivalent subjects  Search modes - Boolean/Phrase | Interface - EBSCOhost Research Databases  Search Screen - Advanced Search  Database - CINAHL | 26,847 |
| S67 | (MH "Practice Guidelines") | Expanders - Apply related words; Apply equivalent subjects  Search modes - Boolean/Phrase | Interface - EBSCOhost Research Databases  Search Screen - Advanced Search  Database - CINAHL | 81,241 |
| S66 | (MH "Consensus") | Expanders - Apply related words; Apply equivalent subjects  Search modes - Boolean/Phrase | Interface - EBSCOhost Research Databases  Search Screen - Advanced Search  Database - CINAHL | 5,025 |
| S65 | (MH "Critical Path") | Expanders - Apply related words; Apply equivalent subjects  Search modes - Boolean/Phrase | Interface - EBSCOhost Research Databases  Search Screen - Advanced Search  Database - CINAHL | 5,541 |
| S64 | S40 AND S63 | Expanders - Apply related words; Apply equivalent subjects  Search modes - Boolean/Phrase | Interface - EBSCOhost Research Databases  Search Screen - Advanced Search  Database - CINAHL | 26 |
| S63 | S47 OR S48 OR S49 OR S50 OR S51 OR S52 OR S53 OR S54 OR S55 OR S56 OR S57 OR S58 OR S59 OR S60 OR S61 OR S62 | Expanders - Apply related words; Apply equivalent subjects  Search modes - Boolean/Phrase | Interface - EBSCOhost Research Databases  Search Screen - Advanced Search  Database - CINAHL | 425,637 |
| S62 | TI trial | Expanders - Apply related words; Apply equivalent subjects  Search modes - Boolean/Phrase | Interface - EBSCOhost Research Databases  Search Screen - Advanced Search  Database - CINAHL | 117,223 |
| S61 | TI control* N2 group# OR AB control* N2 group# | Expanders - Apply related words; Apply equivalent subjects  Search modes - Boolean/Phrase | Interface - EBSCOhost Research Databases  Search Screen - Advanced Search  Database - CINAHL | 124,815 |
| S60 | (MH "Control Group") | Expanders - Apply related words; Apply equivalent subjects  Search modes - Boolean/Phrase | Interface - EBSCOhost Research Databases  Search Screen - Advanced Search  Database - CINAHL | 12,519 |
| S59 | TI control* N2 study OR AB control* N2 study | Expanders - Apply related words; Apply equivalent subjects  Search modes - Boolean/Phrase | Interface - EBSCOhost Research Databases  Search Screen - Advanced Search  Database - CINAHL | 78,448 |
| S58 | (MH "Historically Controlled Study") | Expanders - Apply related words; Apply equivalent subjects  Search modes - Boolean/Phrase | Interface - EBSCOhost Research Databases  Search Screen - Advanced Search  Database - CINAHL | 128 |
| S57 | TI pretest N5 posttest OR AB pretest N5 posttest | Expanders - Apply related words; Apply equivalent subjects  Search modes - Boolean/Phrase | Interface - EBSCOhost Research Databases  Search Screen - Advanced Search  Database - CINAHL | 5,360 |
| S56 | TI "pre-" N5 "post-" OR AB "pre-" N5 "post-" | Expanders - Apply related words; Apply equivalent subjects  Search modes - Boolean/Phrase | Interface - EBSCOhost Research Databases  Search Screen - Advanced Search  Database - CINAHL | 37,850 |
| S55 | (MH "Pretest-Posttest Design") | Expanders - Apply related words; Apply equivalent subjects  Search modes - Boolean/Phrase | Interface - EBSCOhost Research Databases  Search Screen - Advanced Search  Database - CINAHL | 45,983 |
| S54 | TI "time series" OR AB "time series" | Expanders - Apply related words; Apply equivalent subjects  Search modes - Boolean/Phrase | Interface - EBSCOhost Research Databases  Search Screen - Advanced Search  Database - CINAHL | 6,318 |
| S53 | (MH "Nonrandomized Trials") | Expanders - Apply related words; Apply equivalent subjects  Search modes - Boolean/Phrase | Interface - EBSCOhost Research Databases  Search Screen - Advanced Search  Database - CINAHL | 627 |
| S52 | (MH "Interrupted Time Series Analysis") | Expanders - Apply related words; Apply equivalent subjects  Search modes - Boolean/Phrase | Interface - EBSCOhost Research Databases  Search Screen - Advanced Search  Database - CINAHL | 575 |
| S51 | TI ( control* N3 ("before and after" or "before after") ) OR AB ( control* N3 ("before and after" or "before after") ) | Expanders - Apply related words; Apply equivalent subjects  Search modes - Boolean/Phrase | Interface - EBSCOhost Research Databases  Search Screen - Advanced Search  Database - CINAHL | 1,842 |
| S50 | (MH "Controlled Before-After Studies") | Expanders - Apply related words; Apply equivalent subjects  Search modes - Boolean/Phrase | Interface - EBSCOhost Research Databases  Search Screen - Advanced Search  Database - CINAHL | 198 |
| S49 | TI ( nRCT or "non-RCT" ) OR AB ( nRCT or "non-RCT" ) | Expanders - Apply related words; Apply equivalent subjects  Search modes - Boolean/Phrase | Interface - EBSCOhost Research Databases  Search Screen - Advanced Search  Database - CINAHL | 174 |
| S48 | TI ( nonrandom* or (non W0 random*) or (quasi W0 random*) or (quasi W0 experiment*) ) OR AB ( nonrandom* or (non W0 random*) or (quasi W0 random*) or (quasi W0 experiment*) ) | Expanders - Apply related words; Apply equivalent subjects  Search modes - Boolean/Phrase | Interface - EBSCOhost Research Databases  Search Screen - Advanced Search  Database - CINAHL | 23,669 |
| S47 | TI control* N2 trial OR AB control* N2 trial | Expanders - Apply related words; Apply equivalent subjects  Search modes - Boolean/Phrase | Interface - EBSCOhost Research Databases  Search Screen - Advanced Search  Database - CINAHL | 135,712 |
| S46 | S40 AND S45 | Expanders - Apply related words; Apply equivalent subjects  Search modes - Boolean/Phrase | Interface - EBSCOhost Research Databases  Search Screen - Advanced Search  Database - CINAHL | 33 |
| S45 | S41 OR S42 OR S43 OR S44 | Expanders - Apply related words; Apply equivalent subjects  Search modes - Boolean/Phrase | Interface - EBSCOhost Research Databases  Search Screen - Advanced Search  Database - CINAHL | 506,749 |
| S44 | TI trial | Expanders - Apply related words; Apply equivalent subjects  Search modes - Boolean/Phrase | Interface - EBSCOhost Research Databases  Search Screen - Advanced Search  Database - CINAHL | 117,223 |
| S43 | TI ( (singl* or doubl* or trebl* or tripl*) W0 (mask* or blind* or dumm*) ) OR AB ( (singl* or doubl* or trebl* or tripl*) W0 (mask* or blind* or dumm*) ) | Expanders - Apply related words; Apply equivalent subjects  Search modes - Boolean/Phrase | Interface - EBSCOhost Research Databases  Search Screen - Advanced Search  Database - CINAHL | 51,240 |
| S42 | TI ( randomi?ed or randomi?ation# or randomly or RCT or placebo* ) OR AB ( randomi?ed or randomi?ation# or randomly or RCT or placebo* ) | Expanders - Apply related words; Apply equivalent subjects  Search modes - Boolean/Phrase | Interface - EBSCOhost Research Databases  Search Screen - Advanced Search  Database - CINAHL | 344,683 |
| S41 | (MH "Randomized Controlled Trials") or (MH "Clinical Trials+") | Expanders - Apply related words; Apply equivalent subjects  Search modes - Boolean/Phrase | Interface - EBSCOhost Research Databases  Search Screen - Advanced Search  Database - CINAHL | 315,434 |
| S40 | S38 AND S39 | Expanders - Apply related words; Apply equivalent subjects  Search modes - Boolean/Phrase | Interface - EBSCOhost Research Databases  Search Screen - Advanced Search  Database - CINAHL | 632 |
| S39 |  | Limiters - Published Date: 20110101-20211231  Expanders - Apply related words; Apply equivalent subjects  Search modes - Boolean/Phrase | Interface - EBSCOhost Research Databases  Search Screen - Advanced Search  Database - CINAHL | 4,569,422 |
| S38 | S36 NOT S37 | Expanders - Apply related words; Apply equivalent subjects  Search modes - Boolean/Phrase | Interface - EBSCOhost Research Databases  Search Screen - Advanced Search  Database - CINAHL | 865 |
| S37 | (MH "Adult+") NOT ( (MH "Child+") or (MH "Adolescence+") or (MH "Minors") ) | Expanders - Apply related words; Apply equivalent subjects  Search modes - Boolean/Phrase | Interface - EBSCOhost Research Databases  Search Screen - Advanced Search  Database - CINAHL | 1,486,104 |
| S36 | S1 OR S2 OR S3 OR S4 OR S5 OR S6 OR S7 OR S8 OR S9 OR S10 OR S11 OR S12 OR S13 OR S14 OR S15 OR S16 OR S17 OR S18 OR S19 OR S20 OR S21 OR S22 OR S23 OR S24 OR S25 OR S26 OR S27 OR S28 OR S29 OR S30 OR S31 OR S32 OR S33 OR S34 OR S35 | Expanders - Apply related words; Apply equivalent subjects  Search modes - Boolean/Phrase | Interface - EBSCOhost Research Databases  Search Screen - Advanced Search  Database - CINAHL | 1,004 |
| S35 | TI ( (MPS W0 1#) or MPS1# or (MPS W0 I#) or MPSI# or "MPS1-H" or "MPSIH/S" or "MPS1H/S" or "MPS1-HS" or "MPS1-S" or MPSIS or "MPS 2" or MPS2 or "MPS II" or MPSII or (MPS W0 3#) or MPS3# or (MPS W0 III#) or MPSIII# or (MPS W0 4#) or MPS4# or (MPS W0 IV#) or MPSIV# or "MPS 6" or MPS6 or "MPS VI" or MPSVI or "MPS 7" or MPS7 or "MPS VII" or MPSVII or "MPS 9" or MPS9 or "MPS IX" or MPSIX ) OR AB ( (MPS W0 1#) or MPS1# or (MPS W0 I#) or MPSI# or "MPS1-H" or "MPSIH/S" or "MPS1H/S" or "MPS1-HS" or "MPS1-S" or MPSIS or "MPS 2" or MPS2 or "MPS II" or MPSII or (MPS W0 3#) or MPS3# or (MPS W0 III#) or MPSIII# or (MPS W0 4#) or MPS4# or (MPS W0 IV#) or MPSIV# or "MPS 6" or MPS6 or "MPS VI" or MPSVI or "MPS 7" or MPS7 or "MPS VII" or MPSVII or "MPS 9" or MPS9 or "MPS IX" or MPSIX ) | Expanders - Apply related words; Apply equivalent subjects  Search modes - Boolean/Phrase | Interface - EBSCOhost Research Databases  Search Screen - Advanced Search  Database - CINAHL | 266 |
| S34 | TI ( (myxedema* or myxoedema* or myxooedema* or myxo-oedema*) N3 (papular or papulos*) ) OR AB ( (myxedema* or myxoedema* or myxooedema* or myxo-oedema*) N3 (papular or papulos*) ) | Expanders - Apply related words; Apply equivalent subjects  Search modes - Boolean/Phrase | Interface - EBSCOhost Research Databases  Search Screen - Advanced Search  Database - CINAHL | 8 |
| S33 | TI ( (mucinosis or mucinous*) N3 (papular* or papulos*) ) OR AB ( (mucinosis or mucinous*) N3 (papular* or papulos*) ) | Expanders - Apply related words; Apply equivalent subjects  Search modes - Boolean/Phrase | Interface - EBSCOhost Research Databases  Search Screen - Advanced Search  Database - CINAHL | 15 |
| S32 | TI ( lichen N1 (fibromucinoidos* or fibro-mucinoidos* or myxedematos* or myxoedematos* or myxooedematos* or myxo-oedematos*) ) OR AB ( lichen N1 (fibromucinoidos* or fibro-mucinoidos* or myxedematos* or myxoedematos* or myxooedematos* or myxo-oedematos*) ) | Expanders - Apply related words; Apply equivalent subjects  Search modes - Boolean/Phrase | Interface - EBSCOhost Research Databases  Search Screen - Advanced Search  Database - CINAHL | 17 |
| S31 | TI ( scleromyxedem* or (sclero W0 myxedem*) ) OR AB ( scleromyxedem* or (sclero W0 myxedem*) ) | Expanders - Apply related words; Apply equivalent subjects  Search modes - Boolean/Phrase | Interface - EBSCOhost Research Databases  Search Screen - Advanced Search  Database - CINAHL | 67 |
| S30 | TI ( ("beta-glucuronidase" or GUSB) N2 deficien* ) OR AB ( ("beta-glucuronidase" or GUSB) N2 deficien* ) | Expanders - Apply related words; Apply equivalent subjects  Search modes - Boolean/Phrase | Interface - EBSCOhost Research Databases  Search Screen - Advanced Search  Database - CINAHL | 1 |
| S29 | TI ( sly N2 (disease# or syndrome#) ) OR AB ( sly N2 (disease# or syndrome#) ) | Expanders - Apply related words; Apply equivalent subjects  Search modes - Boolean/Phrase | Interface - EBSCOhost Research Databases  Search Screen - Advanced Search  Database - CINAHL | 9 |
| S28 | TI "mckusick 25320" OR AB "mckusick 25320" | Expanders - Apply related words; Apply equivalent subjects  Search modes - Boolean/Phrase | Interface - EBSCOhost Research Databases  Search Screen - Advanced Search  Database - CINAHL | 0 |
| S27 | TI ( ("arylsulfatase b" or "arylsulphatase b" or n-acetylgalactosamine-4-sulfat* or n-acetylgalactosamine-4-sulphat* or ARSB) N2 deficien* ) OR AB ( ("arylsulfatase b" or "arylsulphatase b" or n-acetylgalactosamine-4-sulfat* or n-acetylgalactosamine-4-sulphat* or ARSB) N2 deficien* ) | Expanders - Apply related words; Apply equivalent subjects  Search modes - Boolean/Phrase | Interface - EBSCOhost Research Databases  Search Screen - Advanced Search  Database - CINAHL | 7 |
| S26 | TI ( "maroteaux lamy" N2 (disease# or syndrome#) ) OR AB ( "maroteaux lamy" N2 (disease# or syndrome#) ) | Expanders - Apply related words; Apply equivalent subjects  Search modes - Boolean/Phrase | Interface - EBSCOhost Research Databases  Search Screen - Advanced Search  Database - CINAHL | 28 |
| S25 | TI ( eccentro#steochondrodysplas* or eccentro-osteochondrodysplas* or eccentro-osteo-chondrodysplasia* or eccentroosteo-chondrodysplas* ) OR AB ( eccentro#steochondrodysplas* or eccentro-osteochondrodysplas* or eccentro-osteo-chondrodysplasia* or eccentroosteo-chondrodysplas* ) | Expanders - Apply related words; Apply equivalent subjects  Search modes - Boolean/Phrase | Interface - EBSCOhost Research Databases  Search Screen - Advanced Search  Database - CINAHL | 0 |
| S24 | TI "familial osseous" W0 dystroph* OR AB "familial osseous" W0 dystroph* | Expanders - Apply related words; Apply equivalent subjects  Search modes - Boolean/Phrase | Interface - EBSCOhost Research Databases  Search Screen - Advanced Search  Database - CINAHL | 0 |
| S23 | TI ( (osteochondrodystroph* or osteo-chondrodystroph*) N1 deformans ) OR AB ( (osteochondrodystroph* or osteo-chondrodystroph*) N1 deformans ) | Expanders - Apply related words; Apply equivalent subjects  Search modes - Boolean/Phrase | Interface - EBSCOhost Research Databases  Search Screen - Advanced Search  Database - CINAHL | 0 |
| S22 | TI ( (kerato W0 sulfatur*) or (kerato W0 sulphatur*) or keratosulfatur* or keratosulphatur* ) OR AB ( (kerato W0 sulfatur*) or (kerato W0 sulphatur*) or keratosulfatur* or keratosulphatur* ) | Expanders - Apply related words; Apply equivalent subjects  Search modes - Boolean/Phrase | Interface - EBSCOhost Research Databases  Search Screen - Advanced Search  Database - CINAHL | 0 |
| S21 | TI ( ("galactosamine-6-sulfatase" or "galactosamine-6-sulphatase" or "n-acetylgalactosamine-6-sulfate sulfatase" or "n-acetylgalactosamine-6-sulphate sulphatase" or GALNS) N2 deficien* ) OR AB ( ("galactosamine-6-sulfatase" or "galactosamine-6-sulphatase" or "n-acetylgalactosamine-6-sulfate sulfatase" or "n-acetylgalactosamine-6-sulphate sulphatase" or GALNS) N2 deficien* ) | Expanders - Apply related words; Apply equivalent subjects  Search modes - Boolean/Phrase | Interface - EBSCOhost Research Databases  Search Screen - Advanced Search  Database - CINAHL | 4 |
| S20 | TI ( morquio* N2 (disease# or syndrome#) ) OR AB ( morquio* N2 (disease# or syndrome#) ) | Expanders - Apply related words; Apply equivalent subjects  Search modes - Boolean/Phrase | Interface - EBSCOhost Research Databases  Search Screen - Advanced Search  Database - CINAHL | 88 |
| S19 | TI ( ("n-acetylglucosamine-6-sulfate sulfatase" or "n-acetylglucosamine-6-sulphate sulphatase" or "glucosamine n-acetyl-6-sulfatase" or "glucosamine n-acetyl-6-sulphatase" or GNS) N2 deficien* ) OR AB ("n-acetylglucosamine-6-sulfate sulfatase" or "n-acetylglucosamine-6-sulphate sulphatase" or "glucosamine n-acetyl-6-sulfatase" or "glucosamine n-acetyl-6-sulphatase" or GNS) N2 deficien* ) | Expanders - Apply related words; Apply equivalent subjects  Search modes - Boolean/Phrase | Interface - EBSCOhost Research Databases  Search Screen - Advanced Search  Database - CINAHL | 1 |
| S18 | TI ( ("acetyl-CoA alpha-glucosaminide n-acetyltransferase" or "heparan-alpha-glucosaminide n-acetyltransferase" or HGSNAT) N2 deficien* ) OR AB ( ("acetyl-CoA alpha-glucosaminide n-acetyltransferase" or "heparan-alpha-glucosaminide n-acetyltransferase" or HGSNAT) N2 deficien* ) | Expanders - Apply related words; Apply equivalent subjects  Search modes - Boolean/Phrase | Interface - EBSCOhost Research Databases  Search Screen - Advanced Search  Database - CINAHL | 0 |
| S17 | TI ( (polydystrophic* or poly-dystrophic*) N2 (dwarf* or oligophreni* or oligo-phreni*) ) OR AB ( (polydystrophic* or poly-dystrophic*) N2 (dwarf* or oligophreni* or oligo-phreni*) ) | Expanders - Apply related words; Apply equivalent subjects  Search modes - Boolean/Phrase | Interface - EBSCOhost Research Databases  Search Screen - Advanced Search  Database - CINAHL | 0 |
| S16 | TI ( ("n-acetyl-alpha-d-glucosaminidase" or n-acetylglucosamine-6-sulfat* or n-acetylglucosamine-6-sulphat* or NAGLU) N2 deficien* ) OR AB ( ("n-acetyl-alpha-d-glucosaminidase" or n-acetylglucosamine-6-sulfat* or n-acetylglucosamine-6-sulphat* or NAGLU) N2 deficien* ) | Expanders - Apply related words; Apply equivalent subjects  Search modes - Boolean/Phrase | Interface - EBSCOhost Research Databases  Search Screen - Advanced Search  Database - CINAHL | 0 |
| S15 | TI ( (sanfilippo* or (san W0 filippo*)) N2 deficien* ) OR AB ( (sanfilippo* or (san W0 filippo*)) N2 deficien* ) | Expanders - Apply related words; Apply equivalent subjects  Search modes - Boolean/Phrase | Interface - EBSCOhost Research Databases  Search Screen - Advanced Search  Database - CINAHL | 0 |
| S14 | TI "mckusick 30990" OR AB "mckusick 30990" | Expanders - Apply related words; Apply equivalent subjects  Search modes - Boolean/Phrase | Interface - EBSCOhost Research Databases  Search Screen - Advanced Search  Database - CINAHL | 0 |
| S13 | TI heparitinuri* OR AB heparitinuri* | Expanders - Apply related words; Apply equivalent subjects  Search modes - Boolean/Phrase | Interface - EBSCOhost Research Databases  Search Screen - Advanced Search  Database - CINAHL | 0 |
| S12 | TI ( ("heparan sulfate" or "heparan sulphate" or "heparan sulfamidase" or "heparan sulphamidase" or "heparitin sulfate" or "heparitin sulphate" or "heparan-alpha-glucosaminide n-acetyltransferase") N2 (deficien* or (storage W0 disease#)) ) OR AB ( ("heparan sulfate" or "heparan sulphate" or "heparan sulfamidase" or "heparan sulphamidase" or "heparitin sulfate" or "heparitin sulphate" or "heparan-alpha-glucosaminide n-acetyltransferase") N2 (deficien* or (storage W0 disease#)) ) | Expanders - Apply related words; Apply equivalent subjects  Search modes - Boolean/Phrase | Interface - EBSCOhost Research Databases  Search Screen - Advanced Search  Database - CINAHL | 2 |
| S11 | TI hunter* W0 glossit?s OR AB hunter* W0 glossit?s | Expanders - Apply related words; Apply equivalent subjects  Search modes - Boolean/Phrase | Interface - EBSCOhost Research Databases  Search Screen - Advanced Search  Database - CINAHL | 1 |
| S10 | TI ( Hunter* N2 (disease# or syndrome#) ) OR AB ( Hunter* N2 (disease# or syndrome#) ) | Expanders - Apply related words; Apply equivalent subjects  Search modes - Boolean/Phrase | Interface - EBSCOhost Research Databases  Search Screen - Advanced Search  Database - CINAHL | 128 |
| S9 | TI ( (I2S or iduronate* or "iduronate 2" or sulfoiduronate* or sulfo-iduronate*) N2 deficien* ) OR AB ( (I2S or iduronate* or "iduronate 2" or sulfoiduronate* or sulfo-iduronate*) N2 deficien* ) | Expanders - Apply related words; Apply equivalent subjects  Search modes - Boolean/Phrase | Interface - EBSCOhost Research Databases  Search Screen - Advanced Search  Database - CINAHL | 5 |
| S8 | TI "mckusick 25280" OR AB "mckusick 25280" | Expanders - Apply related words; Apply equivalent subjects  Search modes - Boolean/Phrase | Interface - EBSCOhost Research Databases  Search Screen - Advanced Search  Database - CINAHL | 0 |
| S7 | TI ( lipochondrodystroph* or lipo-chondrodystroph* ) OR AB ( lipochondrodystroph* or lipo-chondrodystroph* ) | Expanders - Apply related words; Apply equivalent subjects  Search modes - Boolean/Phrase | Interface - EBSCOhost Research Databases  Search Screen - Advanced Search  Database - CINAHL | 0 |
| S6 | TI ( chondroosteodysplas* or chondro-osteodysplas* or chondroosteodystroph* or chondro-osteodystroph* or chondroosteoplas* or chondro-osteoplas* or (dysostos?s W0 multiplex) ) OR AB ( chondroosteodysplas* or chondro-osteodysplas* or chondroosteodystroph* or chondro-osteodystroph* or chondroosteoplas* or chondro-osteoplas* or (dysostos?s W0 multiplex) ) | Expanders - Apply related words; Apply equivalent subjects  Search modes - Boolean/Phrase | Interface - EBSCOhost Research Databases  Search Screen - Advanced Search  Database - CINAHL | 30 |
| S5 | TI ( "helmholtz harrington " N2 (disease# or syndrome#) ) OR AB ( "helmholtz harrington " N2 (disease# or syndrome#) ) | Expanders - Apply related words; Apply equivalent subjects  Search modes - Boolean/Phrase | Interface - EBSCOhost Research Databases  Search Screen - Advanced Search  Database - CINAHL | 0 |
| S4 | TI ( (Hurler* or Hurler-Scheie* or Pfaundler-Hurler* or Scheie* or Schleie*) N2 (disease# or syndrome#) ) OR AB ( (Hurler* or Hurler-Scheie* or Pfaundler-Hurler* or Scheie* or Schleie*) N2 (disease# or syndrome#) ) | Expanders - Apply related words; Apply equivalent subjects  Search modes - Boolean/Phrase | Interface - EBSCOhost Research Databases  Search Screen - Advanced Search  Database - CINAHL | 101 |
| S3 | TI ( ("alpha-l-iduronidase" or iduronidase) N2 (disease# or syndrome# or deficien*) ) OR AB ( ("alpha-l-iduronidase" or iduronidase) N2 (disease# or syndrome# or deficien*) ) | Expanders - Apply related words; Apply equivalent subjects  Search modes - Boolean/Phrase | Interface - EBSCOhost Research Databases  Search Screen - Advanced Search  Database - CINAHL | 13 |
| S2 | TI ( mucopolysaccharidos?s or gargolylis* or gargoylis* ) OR AB ( mucopolysaccharidos?s or gargolylis* or gargoylis* ) | Expanders - Apply related words; Apply equivalent subjects  Search modes - Boolean/Phrase | Interface - EBSCOhost Research Databases  Search Screen - Advanced Search  Database - CINAHL | 553 |
| S1 | (MH "Mucopolysaccharidoses+") | Expanders - Apply related words; Apply equivalent subjects  Search modes - Boolean/Phrase | Interface - EBSCOhost Research Databases  Search Screen - Advanced Search  Database - CINAHL | 640 |

**1.3 Grey literature searches**

**ClinicalTrials.gov**

2021 May 26

175 Studies found for: Mucopolysaccharidosis | Child

| Terms | Search Results* | Entire Database** |
| --- | --- | --- |
| Mucopolysaccharidosis | 175 studies | 184 studies |

62 Studies found for: Mucopolysaccharidosis 1 | Child

Also searched for Mucopolysaccharidosis I, Mucopolysaccharidosis type I, and MPS I

| Terms | Search Results* | Entire Database** |
| --- | --- | --- |
| Synonyms | | |
| Mucopolysaccharidosis 1 | 57 studies | 61 studies |
| Mucopolysaccharidosis I | 50 studies | 53 studies |
| MPS I | 32 studies | 34 studies |
| Mucopolysaccharidosis type I | 12 studies | 12 studies |
| Mucopolysaccharidosis | 61 studies | 184 studies |

65 Studies found for: Mucopolysaccharidosis I | Child

*Also searched for Mucopolysaccharidosis type I and MPS I*

| Terms | Search Results* | Entire Database** |
| --- | --- | --- |
| Synonyms | | |
| Mucopolysaccharidosis I | 57 studies | 61 studies |
| MPS I | 32 studies | 34 studies |
| Mucopolysaccharidosis type I | 12 studies | 12 studies |
| Mucopolysaccharidosis 1 | 1 studies | 1 studies |
| Mucopolysaccharidosis | 64 studies | 184 studies |

64 Studies found for: Mucopolysaccharidosis Type I | Child

| Terms | Search Results* | Entire Database** |
| --- | --- | --- |
| Synonyms | | |
| Mucopolysaccharidosis Type I | 57 studies | 61 studies |
| Mucopolysaccharidosis I | 50 studies | 53 studies |
| MPS I | 32 studies | 34 studies |
| Alpha-L-Iduronidase | 4 studies | 5 studies |
| Mucopolysaccharidosis 1 | 1 studies | 1 studies |
| Type I | 13 studies | 3,744 studies |
| Type 1 | 1 studies | 3,455 studies |
| Fitzpatrick Skin Phototype I | -- | 1 studies |

61 Studies found for: Mucopolysaccharidosis Type 1 | Child

| Terms | Search Results* | Entire Database** |
| --- | --- | --- |
| Synonyms | | |
| Mucopolysaccharidosis Type 1 | 57 studies | 61 studies |
| Mucopolysaccharidosis I | 50 studies | 53 studies |
| MPS I | 32 studies | 34 studies |
| Mucopolysaccharidosis type I | 12 studies | 12 studies |
| Mucopolysaccharidosis 1 | 1 studies | 1 studies |
| Type 1 | 13 studies | 3,744 studies |
| Type I | 12 studies | 489 studies |
| Fitzpatrick Skin Phototype I | -- | 1 studies |
| Type | 27 studies | 48,600 studies |
| gamma Sarcoglycan | -- | 1 studies |
| Kind | -- | 58 studies |
| Mucopolysaccharidosis | 60 studies | 184 studies |

17 Studies found for: Mucopolysaccharidosis I S | Child

Also searched for Scheie syndrome and MPS I

| Terms | Search Results* | Entire Database** |
| --- | --- | --- |
| Synonyms | | |
| Mucopolysaccharidosis I S | 17 studies | 19 studies |
| Scheie syndrome | 17 studies | 19 studies |
| Mucopolysaccharidosis I | 17 studies | 61 studies |
| MPS I | 16 studies | 34 studies |
| Mucopolysaccharidosis type I | 3 studies | 12 studies |
| Mucopolysaccharidosis 1 | -- | 1 studies |
| Mucopolysaccharidosis | 17 studies | 184 studies |

39 Studies found for: Mucopolysaccharidosis IH | Child

| Terms | Search Results* | Entire Database** |
| --- | --- | --- |
| Synonyms | | |
| Mucopolysaccharidosis IH | 39 studies | 40 studies |
| Hurler syndrome | 35 studies | 36 studies |
| Hurler disease | 3 studies | 3 studies |
| Mucopolysaccharidosis type IH | 2 studies | 2 studies |
| Mucopolysaccharidosis | 34 studies | 184 studies |

54 Studies found for: Mucopolysaccharidosis Type IH | Child

| Terms | Search Results* | Entire Database** |
| --- | --- | --- |
| Synonyms | | |
| Mucopolysaccharidosis Type IH | 54 studies | 56 studies |
| Hurler syndrome | 35 studies | 36 studies |
| MPS I | 32 studies | 34 studies |
| Hurler disease | 3 studies | 3 studies |
| Mucopolysaccharidosis Ih | 1 studies | 1 studies |
| Dysostosis multiplex | 1 studies | 1 studies |
| Gargoylism | 1 studies | 1 studies |
| Type | 17 studies | 48,600 studies |
| gamma Sarcoglycan | -- | 1 studies |
| Kind | -- | 58 studies |
| Mucopolysaccharidosis | 48 studies | 184 studies |

17 Studies found for: Mucopolysaccharidosis Type I-H/S | Child

Also searched for Hurler-Scheie syndrome, Hurler syndrome, and MPS I

| Terms | Search Results* | Entire Database** |
| --- | --- | --- |
| Synonyms | | |
| Mucopolysaccharidosis Type I-H/S | 16 studies | 17 studies |
| Hurler-Scheie syndrome | 16 studies | 17 studies |
| Mucopolysaccharidosis Type I-H | 17 studies | 55 studies |
| MPS I | 15 studies | 34 studies |
| Hurler syndrome | 13 studies | 36 studies |
| Hurler disease | 1 studies | 3 studies |
| Gargoylism | 1 studies | 1 studies |
| Dysostosis multiplex | -- | 1 studies |
| Mucopolysaccharidosis type IH | -- | 2 studies |
| Type | 5 studies | 48,600 studies |
| gamma Sarcoglycan | -- | 1 studies |
| Kind | -- | 58 studies |
| Mucopolysaccharidosis | 17 studies | 184 studies |

65 Studies found for: Mucopolysaccharidosis II | Child

Also searched for Hunter syndrome, Mucopolysaccharidosis type II, MPS II and more

| Terms | Search Results* | Entire Database** |
| --- | --- | --- |
| Synonyms | | |
| Mucopolysaccharidosis II | 55 studies | 60 studies |
| Hunter syndrome | 40 studies | 43 studies |
| MPS II | 28 studies | 30 studies |
| Mucopolysaccharidosis type II | 10 studies | 11 studies |
| Hunter disease | 7 studies | 7 studies |
| MPS2 | 5 studies | 5 studies |
| Iduronate 2-sulfatase deficiency | 2 studies | 2 studies |
| Mucopolysaccharidosis | 62 studies | 184 studies |

57 Studies found for: Mucopolysaccharidosis 2 | Child

Also searched for Mucopolysaccharidosis II, Hunter syndrome, Mucopolysaccharidosis type II and more

| Terms | Search Results* | Entire Database** |
| --- | --- | --- |
| Synonyms | | |
| Mucopolysaccharidosis 2 | 55 studies | 60 studies |
| Mucopolysaccharidosis II | 48 studies | 52 studies |
| Hunter syndrome | 40 studies | 43 studies |
| MPS II | 28 studies | 30 studies |
| Mucopolysaccharidosis type II | 10 studies | 11 studies |
| Hunter disease | 7 studies | 7 studies |
| MPS2 | 5 studies | 5 studies |
| Iduronate 2-sulfatase deficiency | 2 studies | 2 studies |
| Mucopolysaccharidosis | 54 studies | 184 studies |

63 Studies found for: Mucopolysaccharidosis Type II | Child

| Terms | Search Results* | Entire Database** |
| --- | --- | --- |
| Synonyms | | |
| Mucopolysaccharidosis Type II | 55 studies | 60 studies |
| Mucopolysaccharidosis II | 48 studies | 52 studies |
| Hunter syndrome | 40 studies | 43 studies |
| MPS II | 28 studies | 30 studies |
| Hunter disease | 7 studies | 7 studies |
| MPS2 | 5 studies | 5 studies |
| Iduronate 2-sulfatase deficiency | 2 studies | 2 studies |
| Type II | 11 studies | 9,034 studies |
| Type 2 | -- | 8,501 studies |
| Type | 23 studies | 48,600 studies |
| gamma Sarcoglycan | -- | 1 studies |
| Kind | -- | 58 studies |
| Mucopolysaccharidosis | 60 studies | 184 studies |

1 Study found for: Mucopolysaccharidosis Type II Mild Form | Child

| Terms | Search Results* | Entire Database** |
| --- | --- | --- |
| Synonyms | | |
| Mucopolysaccharidosis Type II Mild Form | -- | 0 studies |
| Mucopolysaccharidosis Type II | 1 studies | 60 studies |
| Hunter syndrome | 1 studies | 43 studies |
| Mucopolysaccharidosis II | 1 studies | 52 studies |
| Hunter disease | 1 studies | 7 studies |
| Iduronate 2-sulfatase deficiency | -- | 2 studies |
| MPS II | -- | 30 studies |
| MPS2 | -- | 5 studies |
| Type II | -- | 9,034 studies |
| Type 2 | -- | 8,501 studies |
| Form | 1 studies | 1,239 studies |
| formation | -- | 464 studies |
| Forming | -- | 46 studies |
| Mild | 1 studies | 4,569 studies |
| Grade 1 | -- | 724 studies |
| mildly | -- | 78 studies |
| Score 2 | -- | 3 studies |
| Type | -- | 48,600 studies |
| gamma Sarcoglycan | -- | 1 studies |
| Kind | -- | 58 studies |
| Mucopolysaccharidosis | 1 studies | 184 studies |

42 Studies found for: Mucopolysaccharidosis III | Child

Also searched for Sanfilippo syndrome

| Terms | Search Results* | Entire Database** |
| --- | --- | --- |
| Synonyms | | |
| Mucopolysaccharidosis III | 38 studies | 38 studies |
| Sanfilippo syndrome | 28 studies | 28 studies |
| Sanfilippo disease | 4 studies | 4 studies |
| Mucopolysaccharidosis type III | 4 studies | 4 studies |
| III | 39 studies | 12,319 studies |
| Third | -- | 777 studies |
| Mucopolysaccharidosis | 39 studies | 184 studies |

42 Studies found for: Mucopolysaccharidosis 3 | Child

Also searched for Mucopolysaccharidosis III and Sanfilippo syndrome.

| Terms | Search Results* | Entire Database** |
| --- | --- | --- |
| Synonyms | | |
| Mucopolysaccharidosis 3 | 38 studies | 38 studies |
| Mucopolysaccharidosis III | 35 studies | 35 studies |
| Sanfilippo syndrome | 28 studies | 28 studies |
| Sanfilippo disease | 4 studies | 4 studies |
| Mucopolysaccharidosis type III | 4 studies | 4 studies |
| Mucopolysaccharidosis | 39 studies | 184 studies |

27 Studies found for: Mucopolysaccharidosis Type IIIA | Child

| Terms | Search Results* | Entire Database** |
| --- | --- | --- |
| Synonyms | | |
| Mucopolysaccharidosis Type IIIA | 27 studies | 27 studies |
| MPS IIIA | 14 studies | 14 studies |
| MPSIII | 10 studies | 10 studies |
| Sanfilippo syndrome type A | 9 studies | 9 studies |
| Sanfilippo A | 4 studies | 4 studies |
| mucopolysaccharidosis type III-A | 2 studies | 2 studies |
| Mucopolysaccharidosis III-A | 1 studies | 1 studies |
| N-Sulfoglucosamine sulfohydrolase | 1 studies | 1 studies |
| SGSH gene | 1 studies | 1 studies |
| IIIA | 16 studies | 1,819 studies |
| Type | 20 studies | 48,600 studies |
| gamma Sarcoglycan | -- | 1 studies |
| Kind | -- | 58 studies |
| Mucopolysaccharidosis | 27 studies | 184 studies |

40 Studies found for: Mucopolysaccharidosis Type III A | Child

Also searched for Mucopolysaccharidosis III, Sanfilippo syndrome, and MPSIII.

| Terms | Search Results* | Entire Database** |
| --- | --- | --- |
| Synonyms | | |
| Mucopolysaccharidosis Type III | 39 studies | 39 studies |
| Mucopolysaccharidosis III | 35 studies | 35 studies |
| Sanfilippo syndrome | 28 studies | 28 studies |
| MPSIII | 10 studies | 10 studies |
| Sanfilippo disease | 4 studies | 4 studies |
| Mucopolysaccharidosis type 3 | 2 studies | 2 studies |
| Type III | 7 studies | 183 studies |
| Type 3 | 2 studies | 101 studies |
| III | 37 studies | 12,319 studies |
| Third | -- | 777 studies |
| Type | 31 studies | 48,600 studies |
| gamma Sarcoglycan | -- | 1 studies |
| Kind | -- | 58 studies |
| Mucopolysaccharidosis | 37 studies | 184 studies |

37 Studies found for: Mucopolysaccharidosis Type 3 A | Child

Also searched for Sanfilippo syndrome, MPSIII, and Type III.

| Terms | Search Results* | Entire Database** |
| --- | --- | --- |
| Synonyms | | |
| Mucopolysaccharidosis Type 3 | 35 studies | 35 studies |
| Sanfilippo syndrome | 28 studies | 28 studies |
| MPSIII | 10 studies | 10 studies |
| Sanfilippo disease | 4 studies | 4 studies |
| Mucopolysaccharidosis type III | 4 studies | 4 studies |
| Type 3 | 7 studies | 183 studies |
| Type III | 5 studies | 84 studies |
| Type | 30 studies | 48,600 studies |
| gamma Sarcoglycan | -- | 1 studies |
| Kind | -- | 58 studies |
| Mucopolysaccharidosis | 34 studies | 184 studies |

17 Studies found for: Mucopolysaccharidosis Type 3 B | Child

| Terms | Search Results* | Entire Database** |
| --- | --- | --- |
| Synonyms | | |
| Mucopolysaccharidosis Type 3 B | 15 studies | 15 studies |
| MPS IIIB | 13 studies | 13 studies |
| Mucopolysaccharidosis type IIIB | 10 studies | 10 studies |
| Sanfilippo syndrome type B | 7 studies | 7 studies |
| Sanfilippo B | 4 studies | 4 studies |
| Mucopolysaccharidosis III-B | 2 studies | 2 studies |
| MPS 3B | 2 studies | 2 studies |
| Sanfilippo syndrome B | 2 studies | 2 studies |
| Mucopolysaccharidosis Type 3 | 14 studies | 35 studies |
| Sanfilippo syndrome | 13 studies | 28 studies |
| MPSIII | 4 studies | 10 studies |
| Sanfilippo disease | 1 studies | 4 studies |
| Mucopolysaccharidosis type III | -- | 4 studies |
| Type 3 | 2 studies | 183 studies |
| Type III | -- | 84 studies |
| Type | 16 studies | 48,600 studies |
| gamma Sarcoglycan | -- | 1 studies |
| Kind | -- | 58 studies |
| Mucopolysaccharidosis | 16 studies | 184 studies |

15 Studies found for: Mucopolysaccharidosis Type III-B | Child

| Terms | Search Results* | Entire Database** |
| --- | --- | --- |
| Synonyms | | |
| Mucopolysaccharidosis Type III-B | 15 studies | 15 studies |
| MPS IIIB | 13 studies | 13 studies |
| Sanfilippo syndrome type B | 7 studies | 7 studies |
| Sanfilippo B | 4 studies | 4 studies |
| Mucopolysaccharidosis III-B | 2 studies | 2 studies |
| MPS 3B | 2 studies | 2 studies |
| Mucopolysaccharidosis Type 3 B | 2 studies | 2 studies |
| Sanfilippo syndrome B | 2 studies | 2 studies |
| III-B | 14 studies | 2,525 studies |
| Type | 14 studies | 48,600 studies |
| gamma Sarcoglycan | -- | 1 studies |
| Kind | -- | 58 studies |
| Mucopolysaccharidosis | 15 studies | 184 studies |

15 Studies found for: Mucopolysaccharidosis Type IIIB | Child

Also searched for Sanfilippo syndrome type B and MPS IIIB.

| Terms | Search Results* | Entire Database** |
| --- | --- | --- |
| Synonyms | | |
| Mucopolysaccharidosis III-B | 15 studies | 15 studies |
| MPS IIIB | 13 studies | 13 studies |
| Mucopolysaccharidosis type IIIB | 10 studies | 10 studies |
| Sanfilippo syndrome type B | 7 studies | 7 studies |
| Sanfilippo B | 4 studies | 4 studies |
| MPS 3B | 2 studies | 2 studies |
| Mucopolysaccharidosis Type 3 B | 2 studies | 2 studies |
| Sanfilippo syndrome B | 2 studies | 2 studies |
| III-B | 14 studies | 2,525 studies |
| Mucopolysaccharidosis | 15 studies | 184 studies |

158 Studies found for: Mucopolysaccharidosis IV | Child

Also searched for Osteochondrodysplasia and Morquio Syndrome.

| Terms | Search Results* | Entire Database** |
| --- | --- | --- |
| Synonyms | | |
| Mucopolysaccharidosis IV | 157 studies | 196 studies |
| osteochondrodysplasia | 147 studies | 186 studies |
| Morquio Syndrome | 10 studies | 11 studies |
| Morquio disease | 5 studies | 6 studies |
| Mucopolysaccharidosis type IV | 2 studies | 2 studies |
| Mucopolysaccharidosis | 28 studies | 184 studies |

162 Studies found for: Mucopolysaccharidosis 4 | Child

| Terms | Search Results* | Entire Database** |
| --- | --- | --- |
| Synonyms | | |
| Mucopolysaccharidosis 4 | 157 studies | 196 studies |
| osteochondrodysplasia | 147 studies | 186 studies |
| Mucopolysaccharidosis IV | 25 studies | 28 studies |
| Morquio Syndrome | 10 studies | 11 studies |
| Morquio disease | 5 studies | 6 studies |
| Mucopolysaccharidosis type IV | 2 studies | 2 studies |
| Mucopolysaccharidosis | 32 studies | 184 studies |

8 Studies found for: Mucopolysaccharidosis Type 4 | Child

Also searched for Morquio disease

| Terms | Search Results* | Entire Database** |
| --- | --- | --- |
| Synonyms | | |
| Mucopolysaccharidosis Type 4 | 7 studies | 8 studies |
| Morquio disease | 5 studies | 6 studies |
| Mucopolysaccharidosis type IV | 2 studies | 2 studies |
| Type 4 | 3 studies | 80 studies |
| Type IV | 2 studies | 39 studies |
| iv types | 1 studies | 6 studies |
| Type | 4 studies | 48,600 studies |
| gamma Sarcoglycan | -- | 1 studies |
| Kind | -- | 58 studies |
| Mucopolysaccharidosis | 8 studies | 184 studies |

3 Studies found for: Mucopolysaccharidosis 4b | Child

| Terms | Search Results* | Entire Database** |
| --- | --- | --- |
| Synonyms | | |
| Mucopolysaccharidosis 4b | 3 studies | 3 studies |
| Morquio B Disease | 3 studies | 3 studies |
| Mucopolysaccharidosis | 3 studies | 184 studies |

158 Studies found for: Mucopolysaccharidosis IV A | Child

Also searched for Osteochondrodysplasia and Morquio Syndrome

| Terms | Search Results* | Entire Database** |
| --- | --- | --- |
| Synonyms | | |
| Mucopolysaccharidosis IV | 157 studies | 196 studies |
| osteochondrodysplasia | 147 studies | 186 studies |
| Morquio Syndrome | 10 studies | 11 studies |
| Morquio disease | 5 studies | 6 studies |
| Mucopolysaccharidosis type IV | 2 studies | 2 studies |
| Mucopolysaccharidosis | 28 studies | 184 studies |

3 Studies found for: Mucopolysaccharidosis IV-B | Child

| Terms | Search Results* | Entire Database** |
| --- | --- | --- |
| Synonyms | | |
| Mucopolysaccharidosis IV-B | 3 studies | 3 studies |
| Morquio B Disease | 3 studies | 3 studies |
| Mucopolysaccharidosis | 3 studies | 184 studies |

17 Studies found for: Mucopolysaccharidosis V | Child

Also searched for Scheie syndrome

| Terms | Search Results* | Entire Database** |
| --- | --- | --- |
| Synonyms | | |
| Mucopolysaccharidosis V | 17 studies | 19 studies |
| Scheie syndrome | 17 studies | 19 studies |
| Mucopolysaccharidosis | 17 studies | 184 studies |

17 Studies found for: Mucopolysaccharidosis Type V | Child

Also searched for Scheie syndrome

| Terms | Search Results* | Entire Database** |
| --- | --- | --- |
| Synonyms | | |
| Mucopolysaccharidosis Type V | 17 studies | 19 studies |
| Scheie syndrome | 17 studies | 19 studies |
| Type | 4 studies | 48,600 studies |
| gamma Sarcoglycan | -- | 1 studies |
| Kind | -- | 58 studies |
| Mucopolysaccharidosis | 17 studies | 184 studies |

2 Studies found for: Mucopolysaccharidosis Type 5 | Child

| Terms | Search Results* | Entire Database** |
| --- | --- | --- |
| Synonyms | | |
| Mucopolysaccharidosis Type 5 | -- | 0 studies |
| Type 5 | -- | 54 studies |
| 5 types | -- | 1 studies |
| Type | 2 studies | 48,600 studies |
| gamma Sarcoglycan | -- | 1 studies |
| Kind | -- | 58 studies |
| Mucopolysaccharidosis | 2 studies | 184 studies |

29 Studies found for: Mucopolysaccharidosis VI | Child

Also searched for Maroteaux Lamy syndrome and MPS VI.

| Terms | Search Results* | Entire Database** |
| --- | --- | --- |
| Synonyms | | |
| Mucopolysaccharidosis VI | 28 studies | 29 studies |
| MPS VI | 18 studies | 19 studies |
| Maroteaux Lamy syndrome | 13 studies | 13 studies |
| Mucopolysaccharidosis type VI | 4 studies | 4 studies |
| Maroteaux-Lamy disease | 1 studies | 1 studies |
| Mucopolysaccharidosis | 29 studies | 184 studies |

30 Studies found for: Mucopolysaccharidosis 6 | Child

Also searched for Mucopolysaccharidosis VI, Maroteaux Lamy syndrome, and MPS VI.

| Terms | Search Results* | Entire Database** |
| --- | --- | --- |
| Synonyms | | |
| Mucopolysaccharidosis 6 | 28 studies | 29 studies |
| Mucopolysaccharidosis VI | 27 studies | 27 studies |
| MPS VI | 18 studies | 19 studies |
| Maroteaux Lamy syndrome | 13 studies | 13 studies |
| Mucopolysaccharidosis type VI | 4 studies | 4 studies |
| Maroteaux-Lamy disease | 1 studies | 1 studies |
| Mucopolysaccharidosis | 30 studies | 184 studies |

28 Studies found for: Mucopolysaccharidosis Type VI | Child

Also searched for Mucopolysaccharidosis VI, Maroteaux Lamy syndrome, and MPS VI

| Terms | Search Results* | Entire Database** |
| --- | --- | --- |
| Synonyms | | |
| Mucopolysaccharidosis Type vi | 28 studies | 29 studies |
| Mucopolysaccharidosis VI | 27 studies | 27 studies |
| MPS VI | 18 studies | 19 studies |
| Maroteaux Lamy syndrome | 13 studies | 13 studies |
| Maroteaux-Lamy disease | 1 studies | 1 studies |
| Type vi | 5 studies | 64 studies |
| Type 6 | -- | 56 studies |
| Type | 10 studies | 48,600 studies |
| gamma Sarcoglycan | -- | 1 studies |
| Kind | -- | 58 studies |
| Mucopolysaccharidosis | 28 studies | 184 studies |

28 Studies found for: Mucopolysaccharidosis Type 6 | Child

Also searched for Mucopolysaccharidosis VI, Maroteaux Lamy syndrome, MPS VI and more.

| Terms | Search Results* | Entire Database** |
| --- | --- | --- |
| Synonyms | | |
| Mucopolysaccharidosis Type 6 | 28 studies | 29 studies |
| Mucopolysaccharidosis VI | 27 studies | 27 studies |
| MPS VI | 18 studies | 19 studies |
| Maroteaux Lamy syndrome | 13 studies | 13 studies |
| Mucopolysaccharidosis type VI | 4 studies | 4 studies |
| Maroteaux-Lamy disease | 1 studies | 1 studies |
| Type 6 | 5 studies | 64 studies |
| Type VI | 5 studies | 9 studies |
| Type | 10 studies | 48,600 studies |
| gamma Sarcoglycan | -- | 1 studies |
| Kind | -- | 58 studies |
| Mucopolysaccharidosis | 28 studies | 184 studies |

12 Studies found for: Mucopolysaccharidosis VII | Child

Also searched for Sly syndrome, MPS VII, and MPS7

| Terms | Search Results* | Entire Database** |
| --- | --- | --- |
| Synonyms | | |
| Mucopolysaccharidosis VII | 12 studies | 13 studies |
| Sly syndrome | 11 studies | 11 studies |
| MPS VII | 7 studies | 8 studies |
| MPS7 | 6 studies | 6 studies |
| Sly Disease | 1 studies | 1 studies |
| Mucopolysaccharidosis type VII | 1 studies | 1 studies |
| VII | 10 studies | 94 studies |
| Mucopolysaccharidosis | 12 studies | 184 studies |

12 Studies found for: Mucopolysaccharidosis 7 | Child

Also searched for Mucopolysaccharidosis VII, Sly syndrome, MPS VII and more

| Terms | Search Results* | Entire Database** |
| --- | --- | --- |
| Synonyms | | |
| Mucopolysaccharidosis 7 | 12 studies | 13 studies |
| Sly syndrome | 11 studies | 11 studies |
| Mucopolysaccharidosis VII | 10 studies | 10 studies |
| MPS VII | 7 studies | 8 studies |
| MPS7 | 6 studies | 6 studies |
| Sly Disease | 1 studies | 1 studies |
| Mucopolysaccharidosis type VII | 1 studies | 1 studies |
| Mucopolysaccharidosis | 12 studies | 184 studies |

12 Studies found for: Mucopolysaccharidosis Type VII | Child

| Terms | Search Results* | Entire Database** |
| --- | --- | --- |
| Synonyms | | |
| Mucopolysaccharidosis Type VII | 12 studies | 13 studies |
| Sly syndrome | 11 studies | 11 studies |
| Mucopolysaccharidosis VII | 10 studies | 10 studies |
| MPS VII | 7 studies | 8 studies |
| MPS7 | 6 studies | 6 studies |
| Mucopolysaccharidosis type 7 | 5 studies | 5 studies |
| Sly Disease | 1 studies | 1 studies |
| VII | 10 studies | 94 studies |
| Type | 7 studies | 48,600 studies |
| gamma Sarcoglycan | -- | 1 studies |
| Kind | -- | 58 studies |
| Mucopolysaccharidosis | 12 studies | 184 studies |

12 Studies found for: Mucopolysaccharidosis Type 7 | Child

Also searched for Sly syndrome and MPS VII

| Terms | Search Results* | Entire Database** |
| --- | --- | --- |
| Synonyms | | |
| Mucopolysaccharidosis Type 7 | 12 studies | 13 studies |
| Sly syndrome | 11 studies | 11 studies |
| MPS VII | 7 studies | 8 studies |
| Mucopolysaccharidosis type VII | 1 studies | 1 studies |
| Type 7 | 5 studies | 17 studies |
| Type | 7 studies | 48,600 studies |
| gamma Sarcoglycan | -- | 1 studies |
| Kind | -- | 58 studies |
| Mucopolysaccharidosis | 12 studies | 184 studies |

1 Study found for: Mucopolysaccharidosis 8 | Child

| Terms | Search Results* |  | Entire Database** |
| --- | --- | --- | --- |
| Mucopolysaccharidosis 8 | -- |  | 0 studies |
| Mucopolysaccharidosis | 1 studies |  | 184 studies |

1 Study found for: Mucopolysaccharidosis type 8 | Child

| Terms | Search Results* | Entire Database** |
| --- | --- | --- |
| Synonyms | | |
| Mucopolysaccharidosis type 8 | -- | 0 studies |
| type | 1 studies | 48,600 studies |
| gamma Sarcoglycan | -- | 1 studies |
| Kind | -- | 58 studies |
| Mucopolysaccharidosis | 1 studies | 184 studies |

5 Studies found for: Mucopolysaccharidosis 9 | Child

| Terms | Search Results* | Entire Database** |
| --- | --- | --- |
| Mucopolysaccharidosis 9 | -- | 0 studies |
| Mucopolysaccharidosis | 5 studies | 184 studies |

1 Study found for: Mucopolysaccharidosis type 9 | Child

| Terms | Search Results* | Entire Database** |
| --- | --- | --- |
| Synonyms | | |
| Mucopolysaccharidosis type 9 | -- | 0 studies |
| type | 1 studies | 48,600 studies |
| gamma Sarcoglycan | -- | 1 studies |
| Kind | -- | 58 studies |
| Mucopolysaccharidosis | 1 studies | 184 studies |

52 Studies found for: Mucopolysaccharidosis x | Child

| Terms | Search Results* | Entire Database** |
| --- | --- | --- |
| Mucopolysaccharidosis x | -- | 0 studies |
| Mucopolysaccharidosis | 52 studies | 184 studies |

2 Studies found for: Mucopolysaccharidosis 10 | Child

| Terms | Search Results* | Entire Database** |
| --- | --- | --- |
| Mucopolysaccharidosis 10 | -- | 0 studies |
| Mucopolysaccharidosis | 2 studies | 184 studies |

2 Studies found for: Mucopolysaccharidosis type 10 | Child

| Terms | Search Results* | Entire Database** |
| --- | --- | --- |
| Synonyms | | |
| Mucopolysaccharidosis type 10 | -- | 0 studies |
| type | 2 studies | 48,600 studies |
| gamma Sarcoglycan | -- | 1 studies |
| Kind | -- | 58 studies |
| Mucopolysaccharidosis | 2 studies | 184 studies |

11 Studies found for: Mucopolysaccharidosis type x | Child

| Terms | Search Results* | Entire Database** |
| --- | --- | --- |
| Synonyms | | |
| Mucopolysaccharidosis type x | -- | 0 studies |
| type | 11 studies | 48,600 studies |
| gamma Sarcoglycan | -- | 1 studies |
| Kind | -- | 58 studies |
| Mucopolysaccharidosis | 11 studies | 184 studies |

No Studies found for: Natowicz syndrome | Child

4 Studies found for: Hyaluronidase deficiency | Child

| Terms | Search Results* | Entire Database** |
| --- | --- | --- |
| Synonyms | | |
| Hyaluronidase deficiency | -- | 0 studies |
| deficiency | 4 studies | 9,477 studies |
| Deficient | -- | 342 studies |
| Hyaluronidase | 4 studies | 85 studies |
| Amphadase | -- | 1 studies |
| Hyaluronoglucosaminidase | -- | 6 studies |
| invasin | -- | 2 studies |
| Vitrase | -- | 7 studies |

**Screening Form**

*Note: Articles were screened by two independent reviewers (dual review).*

| **Phase One: Titles/Abstract Screening** | |
| --- | --- |
| Include | Exclude |
| Population:   - Children (≤18 years) or mixed population (children and adult) diagnosed with MPS where children make up the majority of the population   Study Design:   - Non-animal intervention studies of MPS - Clinical practice guidelines for MPS and other guidelines and recommendation papers related to MPS outcomes | Population:   - Adult population (>18 years) exclusively - Population not focused on MPS   Study Design:   - Animal/in-vitro studies - Non-intervention studies (i.e., observational studies, cross-sectional studies, case reports, case series, etc.)   Additional exclusions:   - Non-English - Published abstract only (e.g., conference proceeding) |
| **Phase Two: Full Text Screening** | |
| Include | Additional Exclusions |
| - If no exclusion criteria are met, include the article | - Abstract or conference proceeding - Adults (>18 years) exclusively - Age group not specified - Age range unclear - Duplicate/already included - Full text not available - No outcomes reported - Irrelevant outcomes reported - Not a guideline by our definition - Not about patients diagnosed with MPS - Not English - Outcomes not reported separately for children - Outcomes not reported separately for MPS - Published before 2011 - Wrong study design - Other (specify) |
