# Supplementary material for: Review of clinical trials and guidelines for children and youth with mucopolysaccharidosis: outcome selection and measurement
Source: Orphanet J Rare Dis. 2024 Oct 23;19:393. doi: 10.1186/s13023-024-03364-x (PMC11520150; doi:10.1186/s13023-024-03364-x)
Supplement: Supplementary file 5 — Additional file 5. [file 13023_2024_3364_MOESM5_ESM.docx]

**ADDITIONAL FILE 5: SUPPLEMENTARY ON-LINE TABLES**

Table S5-1A: Characteristics of included guidelines.

Table S5-1B: Characteristics of included clinical trials and trial registry records.

Table S5-2: Frequency of outcome reporting by year of publication

Table S5-3: Outcome measurement instrument tools used or recommended to measure each outcome.

Table S5-1A: Characteristics of included guidelines.

| **First author last name or trial registry record number** | **MPS subtype** | **Direct industry funding** | **Author affiliation with industry** | **Statement of editorial independence** | **Competing interests recorded/**  **discussed** | **Publication or registration year** | **Country of publication** |
| --- | --- | --- | --- | --- | --- | --- | --- |
| *GUIDELINES (N=18)* | | | | | | | |
| Akyol et al. (1) | MPS IVA | Yes | No | Yes | Recorded and discussed | 2019 | South Africa |
| Akyol et al. (2) | MPS VI | Yes | No | Yes | Recorded and discussed | 2019 | Brazil |
| Charrow et al. (3) | MPS IVA | Yes | Yes | No | Recorded | 2015 | United States of America |
| Fahnehjelm et al. (4) | MPS (general) | Yes | No | No | Recorded | 2012 | Sweden |
| Ghosh et al. (5) | MPS IIIA-D | No | Yes | No | Recorded | 2017 | United Kingdom |
| Giugliani et al. (6) | MPS II | Yes | No | Yes | Recorded | 2014 | Brazil |
| González-Gutiérrez-Solana et al. (7) | MPS II | No | No | No | Recorded (none declared) | 2018 | Spain |
| Hendriksz et al. (8) | MPS IVA | Yes | No | No | Recorded | 2015 | United Kingdom |
| Kuiper et al. (9) | MPS I-H, MPS I-HS, MPS I-S | No | No | No | Recorded (none declared) | 2019 | The Netherlands |
| Langereis et al. (10) | MPS I-H | No | No | No | Recorded (none declared) | 2013 | The Netherlands |
| McBride et al. (11) | MPS II | No | No | No | Recorded and discussed | 2020 | United States of America |
| Muenzer et al. (12) | MPS II | Yes | Yes | Yes | Recorded | 2012 | United States of America |
| Politei et al. (13) | MPS (general) | Yes | Yes | No | Recorded | 2018 | Argentina |
| Scarpa et al. (14) | MPS II | Yes | No | No | Recorded | 2011 | United Kingdom |
| Solanki et al. (15) | MPS VI | Yes | No | No | Recorded | 2012 | United Kingdom |
| Solano et al. (16) | MPS I-H, MPS I-HS, MPS I-S, MPS II, MPS IVA, MPS VI | Yes | No | No | Recorded | 2020 | Brazil |
| van der Lee et al. (17) | MPS (general) | Yes | Yes | Yes | Recorded | 2020 | United States of America |
| Wang et al. (18) | MPS I-H, MPS I-HS, MPS I-S, MPS II, MPS VI | Yes | No | Yes | Recorded | 2011 | United States of America |
| Table S5-1B: Characteristics of included clinical trials and trial registry records. | | | | | | | |
| *CLINICAL TRIALS AND TRIAL REGISTRY RECORDS (N=55)* | | | | | | | |
| **First author last name or trial registry record number** | **MPS subtype** | **Population** | **Sample size** | **Intervention type** | | **Publication or registration year** | **Country of publication** |
| Berger et al. (19) | MPS IVA | Children and adults | 15 | ERT | | 2017 | United States of America |
| Burton et al. (20) | MPS IVA | Children and adults | 25 | ERT | | 2015 | United States of America |
| Treadwell et al. (21) | MPS IVA | Children and adults | 25 | ERT | | 2017 | United States of America |
| Braunlin et al. (22) | MPS VI | Children and adults | 54 | ERT | | 2013 | United States of America |
| But et al. (23) | MPS VI | Children (<19 years) | 2 | ERT | | 2011 | China |
| Delgadillo et al. (24) | MPS IIIA-C | Children and adults | 19 | SRT | | 2011 | Spain |
| Garcia et al. (25) | MPS VI | Children (<19 years) | 3 | ERT | | 2021 | United States of America |
| Harmatz et al. (26) | MPS VI | Children (<19 years) | 4 | ERT | | 2014 | United States of America |
| Giugliani et al. (27) | MPS I-H | Children (<19 years) | 7 | ERT | | 2017 | The Netherlands |
| Giugliani et al. (28) | MPS I-H, MPS I-HS | Children (<19 years) | 11 | ERT | | 2018 | United States of America |
| Giugliani et al. (29) | MPS II | Children and adults | 19 | ERT | | 2021 | Japan |
| Giugliani et al. (30) | MPS II | Children (<19 years) | 28 | ERT | | 2013 | Brazil |
| Guffon et al. (31) | MPS IIIA-D | Children (<19 years) | 25 | SRT | | 2011 | France |
| Haller et al. (32) | MPS VII | Children and adults | 12 | ERT | | 2019 | United States of America |
| Wang et al. (33) | MPS VII | Children and adults | 12 | ERT | | 2020 | United States of America |
| Harmatz et al. (34) | MPS IVA | Children and adults | 10 | ERT | | 2017 | United States of America |
| Hendriksz et al. (35) | MPS IVA | Children and adults | 176 | ERT | | 2014 | United Kingdom |
| Hendriksz et al. (36) | MPS IVA | Children and adults | 176 | ERT | | 2015 | United Kingdom |
| Hendriksz et al. (37) | MPS IVA | Children and adults | 173 | ERT | | 2016 | United Kingdom |
| Jones et al. (38) | MPS IVA | Children (<19 years) | 15 | ERT | | 2015 | United Kingdom |
| Jones et al. (39) | MPS IIIA | Children and adults | 12 | ERT | | 2016 | The Netherlands |
| Kim et al. (40) | MPS II | Children and adults | 34 | ERT | | 2013 | Korea |
| Kim et al. (41) | MPS II, MPS III (unspecified) | Children (<19 years) | 22 | SRT | | 2013 | United States of America |
| Lund et al. (42) | MPS I-H | Children (<19 years) | 10 | ERT | | 2019 | United States of America |
| Polgreen et al. (43) | MPS I-H | Children (<19 years) | 10 | ERT | | 2020 | United States of America |
| Marucha et al. (44) | MPS II | Children and adults | 7 | SRT | | 2011 | Poland |
| Muenzer et al. (45) | MPS II | Children (<19 years) | 16 | ERT | | 2016 | United States of America |
| EUCTR2011-000212-25-GB (46) | MPS II | N/A | 15 | ERT | | 2011 | N/A |
| Muenzer et al. (47) | MPS II | Children and adults | 94 | ERT | | 2011 | United States of America |
| Okuyama et al. (48) | MPS II | Children (<19 years) | 28 | ERT | | 2021 | Japan |
| Polgreen et al. (49) | MPS I-H, MPS II | Children (<19 years) | 2 | TNF-α inhibitor | | 2017 | United States of America |
| Seo et al. (50) | MPS II | Children (<19 years) | 6 | ERT | | 2021 | Japan |
| Sohn et al. (51) | MPS II | Children (<19 years) | 6 | ERT | | 2015 | Korea |
| Tardieu et al. (52) | MPS IIIA | Children (<19 years) | 4 | Gene therapy | | 2014 | France |
| Tardieu et al. (53) | MPS IIIB | Children (<19 years) | 4 | Gene therapy | | 2017 | France |
| Whitley et al. (54) | MPS IIIB | Children (<19 years) | 11 | ERT | | 2019 | United States of America |
| Wijburg et al. (55) | MPS IIIA | Children (<19 years) | 21 | ERT | | 2019 | United States of America |
| EUCTR2014-003960-20-GB (56) | MPS IIIA | N/A | 17 | ERT | | 2015 | N/A |
| EUCTR2013-001479-18-GB (57) | MPS IIIA-C | N/A | 24 | SRT | | 2014 | N/A |
| NCT03488394 (58) | MPS I-H | N/A | 8 | Gene therapy | | 2018 | N/A |
| NCT04284254 (59) | MPS I-H | N/A | 36 | Gene therapy | | 2020 | N/A |
| NCT02055118 (60) | MPS II | N/A | 58 | ERT | | 2014 | N/A |
| NCT02412787 (61) | MPS II | N/A | 49 | ERT | | 2015 | N/A |
| NCT02455622 (62) | MPS II | N/A | 21 | ERT | | 2015 | N/A |
| NCT03566043 (63) | MPS II | N/A | 18 | Gene therapy | | 2018 | N/A |
| NCT04251026 (64) | MPS II | N/A | 30 | ERT | | 2020 | N/A |
| NCT04571970 (65) | MPS II | N/A | 6 | Gene therapy | | 2020 | N/A |
| NCT04573023 (66) | MPS II | N/A | 50 | ERT | | 2020 | N/A |
| NCT03423186 (67) | MPS IIIA | N/A | 6 | ERT | | 2018 | N/A |
| NCT03811028 (68) | MPS IIIA | N/A | 6 | ERT | | 2019 | N/A |
| NCT04088734 (69) | MPS IIIA | N/A | 12 | Gene therapy | | 2019 | N/A |
| NCT04201405 (70) | MPS IIIA | N/A | 3 to 5 | Gene therapy | | 2019 | N/A |
| NCT02754076 (71) | MPS IIIB | N/A | 23 | ERT | | 2016 | N/A |
| NCT03784287 (72) | MPS IIIB | N/A | 20 | ERT | | 2018 | N/A |
| NCT02418455 (73) | MPS VII | N/A | 8 | ERT | | 2015 | N/A |

ERT: enzyme replacement therapy; MPS: mucopolysaccharidosis; SRT: substrate reduction therapy; TNF-α: tumor necrosis factor alpha.

Table S5-2: Frequency of outcome reporting by year of publication^a^.

| **Outcome** | **# (%) of all studies (n=61)** | **# (%) of studies, 2011-2014**  **(n=22)** | **# (%) of studies, 2015-2018 (n=24)** | **# (%) of studies, 2019-2021**  **(n=21)** |
| --- | --- | --- | --- | --- |
| **LIFE IMPACT** | **44 (72%)** | **16 (73%)** | **17 (71%)** | **16 (76%)** |
| *Domain: Behaviour and emotional health* | *13 (21%)* | *6 (27%)* | *3 (12%)* | *4 (19%)* |
| Mood and behaviour changes | 13 (21%) | 6 (27%) | 3 (12%) | 4 (19%) |
| *Domain: Child and caregiver/family support* | *40 (66%)* | *13 (59%)* | *17 (71%)* | *14 (67%)* |
| Activities of daily living | 30 (49%) | 7 (32%) | 13 (54%) | 13 (62%) |
| Quality of life | 24 (39%) | 7 (32%) | 8 (33%) | 10 (48%) |
| Pain | 13 (21%) | 1 (5%) | 6 (25%) | 6 (29%) |
| Sleep apnoea/sleep disordered breathing | 10 (16%) | 4 (18%) | 3 (12%) | 3 (14%) |
| Caregiver/family impact | 6 (10%) | 1 (5%) | 2 (8%) | 4 (19%) |
| Fatigue | 3 (5%) | 0 (0%) | 0 (0%) | 3 (14%) |
| Sleep disturbances | 2 (3%) | 1 (5%) | 0 (0%) | 1 (5%) |
| Autonomy/independence | 1 (2%) | 1 (5%) | 0 (0%) | 0 (0%) |
| Overall health (parent assessed) | 1 (2%) | 0 (0%) | 0 (0%) | 1 (5%) |
| Social impact and function | 1 (2%) | 0 (0%) | 0 (0%) | 1 (5%) |
| *Domain: Mobility and strength* | *24 (39%)* | *10 (45%)* | *8 (33%)* | *8 (38%)* |
| Endurance/exercise capacity | 14 (23%) | 4 (18%) | 5 (21%) | 6 (29%) |
| Mobility | 14 (23%) | 5 (23%) | 5 (21%) | 5 (24%) |
| Grip strength | 5 (8%) | 0 (0%) | 3 (12%) | 2 (10%) |
| Pinch strength | 3 (5%) | 0 (0%) | 2 (8%) | 1 (5%) |
| Strength | 3 (5%) | 1 (5%) | 1 (4%) | 1 (5%) |
| Need for mobility aid | 2 (3%) | 0 (0%) | 0 (0%) | 2 (10%) |
| **GROWTH AND DEVELOPMENT** | **47 (77%)** | **13 (59%)** | **20 (83%)** | **19 (90%)** |
| *Domain: Physical growth and anthropometry* | *27 (44%)* | *8 (36%)* | *12 (50%)* | *9 (43%)* |
| Height/length | 18 (30%) | 6 (27%) | 10 (42%) | 4 (19%) |
| Weight | 16 (26%) | 5 (23%) | 8 (33%) | 5 (24%) |
| Growth | 15 (25%) | 5 (23%) | 5 (21%) | 6 (29%) |
| Head circumference | 10 (16%) | 4 (18%) | 4 (17%) | 2 (10%) |
| Puberty | 3 (5%) | 0 (0%) | 1 (4%) | 2 (10%) |
| Arm span | 2 (3%) | 0 (0%) | 1 (4%) | 1 (5%) |
| *Domain: Cognition and development* | *36 (59%)* | *10 (45%)* | *13 (54%)* | *17 (81%)* |
| Cognitive function and early development | 29 (48%) | 8 (36%) | 11 (46%) | 13 (62%) |
| Fine-motor ability | 5 (8%) | 1 (5%) | 2 (8%) | 3 (14%) |
| Speech abilities | 5 (8%) | 3 (14%) | 0 (0%) | 2 (10%) |
| School performance | 1 (2%) | 0 (0%) | 1 (4%) | 0 (0%) |
| **PATHOPHYSIOLOGICAL MANIFESTATIONS** | **60 (98%)** | **22 (100%)** | **23 (96%)** | **21 (100%)** |
| *Domain: Adverse events/effects* | *51 (84%)* | *17 (77%)* | *20 (83%)* | *19 (90%)* |
| General adverse events^b^ | 45 (74%) | 14 (64%) | 18 (75%) | 18 (86%) |
| Immune-related adverse events^b^ | 39 (64%) | 9 (41%) | 18 (75%) | 15 (71%) |
| *Domain: Blood and lymphatic system outcomes* | *22 (36%)* | *9 (41%)* | *10 (42%)* | *5 (24%)* |
| General haematology | 20 (33%) | 8 (36%) | 10 (42%) | 4 (19%) |
| Biochemical evaluations | 6 (10%) | 3 (14%) | 1 (4%) | 2 (10%) |
| Hypoglycemia | 1 (2%) | 0 (0%) | 1 (4%) | 0 (0%) |
| *Domain: Cardiac outcomes* | *32 (52%)* | *11 (50%)* | *14 (58%)* | *11 (52%)* |
| Cardiac function | 32 (52%) | 11 (50%) | 14 (58%) | 11 (52%) |
| *Domain: Congenital, familial and genetic outcomes* | *36 (59%)* | *0 (0%)* | *2 (8%)* | *3 (14%)* |
| Gene therapy monitoring outcomes | 4 (7%) | 0 (0%) | 2 (8%) | 2 (10%) |
| Successful donor chimerism | 2 (3%) | 0 (0%) | 0 (0%) | 2 (10%) |
| *Domain: Ear and labyrinth outcomes* | *12 (20%)* | *7 (32%)* | *3 (12%)* | *4 (19%)* |
| Hearing | 10 (16%) | 5 (23%) | 3 (12%) | 4 (19%) |
| Ear, nose, and throat manifestations | 5 (8%) | 3 (14%) | 0 (0%) | 2 (10%) |
| *Domain: Endocrine outcomes* | *2 (3%)* | *1 (5%)* | *0 (0%)* | *1 (5%)* |
| Endocrine function | 1 (2%) | 0 (0%) | 0 (0%) | 1 (5%) |
| Thyroid function | 1 (2%) | 1 (5%) | 0 (0%) | 0 (0%) |
| *Domain: Eye outcomes* | *11 (18%)* | *5 (23%)* | *3 (12%)* | *5 (24%)* |
| Vision/eye health | 11 (18%) | 5 (23%) | 3 (12%) | 5 (24%) |
| *Domain: Gastrointestinal outcomes* | *3 (5%)* | *1 (5%)* | *1 (4%)* | *1 (5%)* |
| Gastrointestinal manifestations | 3 (5%) | 1 (5%) | 1 (4%) | 1 (5%) |
| *Domain: General outcomes* | *45 (74%)* | *16 (73%)* | *15 (62%)* | *16 (76%)* |
| Overall health (clinician assessment) | 24 (39%) | 11 (50%) | 8 (33%) | 6 (29%) |
| Organomegaly | 24 (39%) | 8 (36%) | 5 (21%) | 11 (52%) |
| Vital signs | 23 (38%) | 7 (32%) | 9 (38%) | 8 (38%) |
| Surgical intervention required^c^ | 9 (15%) | 4 (18%) | 1 (4%) | 4 (19%) |
| Changes to concomitant medications | 8 (13%) | 3 (14%) | 3 (12%) | 2 (10%) |
| Disease progression | 7 (11%) | 4 (18%) | 1 (4%) | 2 (10%) |
| Oral health | 5 (8%) | 2 (9%) | 1 (4%) | 2 (10%) |
| Facial features | 4 (7%) | 3 (14%) | 1 (4%) | 0 (0%) |
| Changes relative to personalized treatment goals | 1 (2%) | 0 (0%) | 0 (0%) | 1 (5%) |
| Dentition | 1 (2%) | 1 (5%) | 0 (0%) | 0 (0%) |
| Hair morphology | 1 (2%) | 1 (5%) | 0 (0%) | 0 (0%) |
| Short neck | 1 (2%) | 1 (5%) | 0 (0%) | 0 (0%) |
| *Domain: Hepatobiliary outcomes* | *4 (7%)* | *2 (9%)* | *1 (4%)* | *1 (5%)* |
| Liver function | 4 (7%) | 2 (9%) | 1 (4%) | 1 (5%) |
| *Domain: Infection and infestation outcomes* | *3 (5%)* | *1 (5%)* | *1 (4%)* | *1 (5%)* |
| Infections (non-specific) | 3 (5%) | 1 (5%) | 1 (4%) | 1 (5%) |
| *Domain: Metabolism and nutrition outcomes* | *46 (75%)* | *13 (59%)* | *21 (88%)* | *17 (81%)* |
| Urinary glycosaminoglycans (GAGs) | 38 (62%) | 10 (45%) | 17 (71%) | 14 (67%) |
| Cerebrospinal fluid (CSF) GAGs | 18 (30%) | 2 (9%) | 8 (33%) | 10 (48%) |
| Drug therapy monitoring (CSF/blood) | 9 (15%) | 4 (18%) | 5 (21%) | 3 (14%) |
| Blood GAGs | 8 (13%) | 1 (5%) | 2 (8%) | 6 (29%) |
| Enzyme activity | 8 (13%) | 0 (0%) | 4 (17%) | 4 (19%) |
| *Domain: Musculoskeletal and connective tissue outcomes* | *29 (48%)* | *12 (55%)* | *10 (42%)* | *9 (43%)* |
| Upper limb joint function/range of motion (ROM) | 13 (21%) | 3 (14%) | 4 (17%) | 6 (29%) |
| Lower limb joint function/ROM | 12 (20%) | 3 (14%) | 3 (12%) | 6 (29%) |
| Bone health^d^ | 10 (16%) | 5 (23%) | 6 (25%) | 1 (5%) |
| Joint mobility/ROM (general) | 10 (16%) | 3 (14%) | 3 (12%) | 4 (19%) |
| Hernia | 5 (8%) | 2 (9%) | 1 (4%) | 2 (10%) |
| Hip abnormalities | 4 (7%) | 1 (5%) | 1 (4%) | 2 (10%) |
| Neuromuscular manifestations | 4 (7%) | 3 (14%) | 1 (4%) | 0 (0%) |
| Carpal tunnel syndrome | 3 (5%) | 2 (9%) | 0 (0%) | 1 (5%) |
| Hand joint mobility/ ROM | 3 (5%) | 2 (9%) | 0 (0%) | 1 (5%) |
| Valgus deformity of lower limbs | 3 (5%) | 0 (0%) | 1 (4%) | 2 (10%) |
| Arthropathy | 2 (3%) | 2 (9%) | 0 (0%) | 0 (0%) |
| *Domain: Nervous system outcomes* | *30 (49%)* | *8 (36%)* | *12 (50%)* | *12 (57%)* |
| General neurological manifestations | 28 (46%) | 7 (32%) | 12 (50%) | 11 (52%) |
| Spinal manifestations | 11 (18%) | 4 (18%) | 3 (12%) | 4 (19%) |
| CSF pressure | 2 (3%) | 0 (0%) | 1 (4%) | 1 (5%) |
| Seizures (including due to epilepsy) | 2 (3%) | 2 (9%) | 0 (0%) | 0 (0%) |
| *Domain: Renal and urinary outcomes* | *17 (28%)* | *7 (32%)* | *6 (25%)* | *5 (24%)* |
| Urinalysis | 14 (23%) | 4 (18%) | 6 (25%) | 5 (24%) |
| Renal function | 3 (5%) | 2 (9%) | 0 (0%) | 1 (5%) |
| Urinary manifestations | 1 (2%) | 1 (5%) | 0 (0%) | 0 (0%) |
| *Domain: Respiratory, thoracic and mediastinal outcomes* | *17 (28%)* | *5 (23%)* | *6 (25%)* | *7 (33%)* |
| Lung function | 16 (26%) | 4 (18%) | 6 (25%) | 7 (33%) |
| Airway manifestations | 6 (10%) | 2 (9%) | 2 (8%) | 2 (10%) |
| *Domain: Skin and subcutaneous tissue outcomes* | *1 (2%)* | *1 (5%)* | *0 (0%)* | *0 (0%)* |
| Skin manifestations | 1 (2%) | 1 (5%) | 0 (0%) | 0 (0%) |
| *Domain: Vascular outcomes* | *1 (2%)* | *1 (5%)* | *0 (0%)* | *0 (0%)* |
| Hypertension | 1 (2%) | 1 (5%) | 0 (0%) | 0 (0%) |
| **HEALTH RESOURCE USE** | **2 (3%)** | **2 (9%)** | **0 (0%)** | **0 (0%)** |
| *Domain: Health resource use* | 2 (3%) | 2 (9%) | 0 (0%) | 0 (0%) |
| Health resource use | 2 (3%) | 2 (9%) | 0 (0%) | 0 (0%) |
| **DEATH** | **3 (5%)** | **1 (5%)** | **1 (4%)** | **1 (5%)** |
| *Domain: Life expectancy* | 3 (5%) | 1 (5%) | 1 (4%) | 1 (5%) |
| Life expectancy | 3 (5%) | 1 (5%) | 1 (4%) | 1 (5%) |

CSF: cerebrospinal fluid; GAGs: glycosaminoglycans; MPS: mucopolysaccharidosis; ROM: range of motion.

^a^ Year ranges are not mutually exclusive. When articles pertaining to the same study were published in different years, we counted the outcomes as being reported in all applicable year ranges.

^b^ General adverse events were extracted as an outcome if they were reported in the methods or the results of a trial. Immune-related adverse events were only extracted if they were mentioned in the methods so as to not distort the findings based on the observed safety profile of the evaluated interventions.

^c^Surgical intervention required captures both broad assessments (e.g., surgical procedures) and those related to specific manifestations (e.g., need for kyphosis surgery).

^d^Bone health captures both structural changes (e.g., cervical spine MRI, abnormal bone thickness and shape) and changes to bone composition (e.g., bone density, bone and cartilage metabolism).

Table S5-3: Outcome measurement instrument tools used or recommended to measure each outcome.

| **Outcome and # of studies reporting or recommending the outcome** | **# of unique instruments** | **Outcome measurement instrument^a^** | **# (%) of studies^b^** |
| --- | --- | --- | --- |
| ***CORE AREA: LIFE IMPACT*** | | | |
| Mood and behaviour changes (n=13 studies reported this outcome) | 15 | *Sanfilippo Behaviour Rating Scale (74) | 3 (23%) |
|  |  | ^#^Vineland Adaptive Behavior Scales (75) | 2 (15%) |
|  |  | *MPS III Disability Scale (investigator-developed) (24) | 1 (8%) |
|  |  | Test of Variables of Attention (TOVA) (76) | 1 (8%) |
|  |  | Conners scale (77) | 1 (8%) |
|  |  | Conners continuous performance test (78) | 1 (8%) |
|  |  | Toddler Behaviour Assessment Questionnaire (79) | 1 (8%) |
|  |  | Child Health Questionnaire (CHQ) (80) | 1 (8%) |
|  |  | *Hunter Behavior Rating Scale (81) | 1 (8%) |
|  |  | Child Behavior Checklist (CBCL, brief) (82) | 1 (8%) |
|  |  | Behavior Assessment System for Children (BASC) (83) | 1 (8%) |
|  |  | Behavior Rating Inventory of Executive Function (BRIEF) (84) | 1 (8%) |
|  |  | Bayley Scales of Infant and Toddler Development (85) | 1 (8%) |
|  |  | ^#^Kaufman Assessment Battery for Children (86) | 1 (8%) |
|  |  | Mullen Scales of Early Learning (MSEL) (87) | 1 (8%) |
|  |  | General, unclear, or not specified | 7 (54%) |
| Activities of daily living^c^ (n=30) | 14 | ^#^Vineland Adaptive Behavior Scales (75) | 15 (50%) |
|  |  | *MPS Health Assessment Questionnaire (MPS HAQ) (88) | 5 (17%) |
|  |  | ^#^Childhood Health Assessment Questionnaire (CHAQ) (89) | 3 (10%) |
|  |  | Six-minute walk test (6MWT) (90) | 3 (10%) |
|  |  | Timed 25-foot walk test (T25FW) (91) | 3 (10%) |
|  |  | Denver Developmental Screening Test (92) | 2 (7%) |
|  |  | Bayley Scales of Infant and Toddler Development (85) | 1 (3%) |
|  |  | ^#^Kaufman Assessment Battery for Children (86) | 1 (3%) |
|  |  | Brunet-Lezine Revised Test (93) | 1 (3%) |
|  |  | Child Health Questionnaire (CHQ) (80) | 1 (3%) |
|  |  | *Toileting Abilities Survey (94) | 1 (3%) |
|  |  | ^#^Functional Dexterity Test (95) | 1 (3%) |
|  |  | Functional Independence Measure (FIM) (96) | 1 (3%) |
|  |  | Pediatric Evaluation of Disabilities Inventory (PEDI) (97) | 1 (3%) |
|  |  | Mullen Scales of Early Learning (MSEL) (87) | 1 (3%) |
|  |  | General, unclear, or not specified | 10 (33%) |
| Quality of life (n=24) | 13 | EQ-5D-5L (98) | 6 (25%) |
|  |  | Pediatric Quality of Life Inventory (PedsQL) (99) | 5 (21%) |
|  |  | *Hunter Syndrome-Functional Outcomes for Clinical Understanding Scale (HS-FOCUS) (100) | 4 (17%) |
|  |  | ^#^Short Form 36 Health Survey Questionnaire (SF-36) (101) | 4 (17%) |
|  |  | Child Health Questionnaire (CHQ) (80) | 3 (13%) |
|  |  | ^#^Childhood Health Assessment Questionnaire (CHAQ) (89) | 2 (8%) |
|  |  | *MPS Health Assessment Questionnaire (MPS HAQ) (88) | 2 (8%) |
|  |  | Infant Toddler Quality of Life Questionnaire (IT-QOL) (102) | 2 (8%) |
|  |  | ^#^Vineland Adaptive Behavior Scales (75) | 1 (4%) |
|  |  | Pediatrics Outcomes Data Collection Instrument (PODCI) (103) | 1 (4%) |
|  |  | Children's Sleep Habits Questionnaire (CSHQ) (104) | 1 (4%) |
|  |  | Health Utilities Index (HUI) (105) | 1 (4%) |
|  |  | KIDSCREEN Questionnaire (106) | 1 (4%) |
|  |  | General, unclear, or not specified | 7 (29%) |
| Pain (n=13) | 10 | ^#^Adolescent Pediatric Pain Tool (APPT) (107) | 2 (15%) |
|  |  | Faces Pain Scale-Revised (FPS-R) (108) | 2 (15%) |
|  |  | Brief Pain Inventory Short Form (BPI-SF) (109) | 2 (15%) |
|  |  | ^#^Childhood Health Assessment Questionnaire (CHAQ) (89) | 1 (8%) |
|  |  | EQ-5D-5L (98) | 1 (8%) |
|  |  | Face, Legs, Activity, Cry and Consolability Scale (FLACC) (110) | 1 (8%) |
|  |  | Numeric Rating Scale (NRS) (111) | 1 (8%) |
|  |  | ^#^Short Form 36 Health Survey Questionnaire (SF-36) (101) | 1 (8%) |
|  |  | Child Health Questionnaire (CHQ) (80) | 1 (8%) |
|  |  | Pediatric Pain Questionnaire (PPQ) (112) | 1 (8%) |
|  |  | General, unclear, or not specified | 11 (85%) |
| Caregiver/family impact (n=6) | 7 | Pediatric Quality of Life Inventory (PedsQL) (Family Impact Module) (113) | 3 (50%) |
|  |  | Parenting Stress Index (114) | 2 (33%) |
|  |  | Caregiver Impact Questionnaire (CIQ) (115) | 1 (17%) |
|  |  | Child Health Questionnaire (CHQ) (80) | 1 (17%) |
|  |  | Infant Toddler Quality of Life Questionnaire (IT-QoL) (102) | 1 (17%) |
|  |  | Zarit Burden Interview (ZBI) (116) | 1 (17%) |
|  |  | Beck Depression Inventory (BDI) (117) | 1 (17%) |
|  |  | General, unclear, or not specified | 2 (33%) |
| Fatigue (n=3) | 1 | Pediatric Quality of Life Inventory (PedsQL) (Multidimensional Fatigue Scale) (118) | 1 (33%) |
|  |  | General, unclear, or not specified | 3 (100%) |
| Sleep disturbances (n=2) | 2 | Children’s Sleep Habits Questionnaire (CSHQ) (104,119) | 1 (50%) |
|  |  | *Sleep Disturbances in Sanfilippo Syndrome (120) | 1 (50%) |
| Social impact and function (n=1) | 3 | Behavior Assessment System for Children (BASC) (83) | 1 (100%) |
|  |  | Behavior Rating Inventory of Executive Function (BRIEF) (84) | 1 (100%) |
|  |  | Child Behavior Checklist (CBCL, brief) (82) | 1 (100%) |
| Autonomy/independence (n=1) | 2 | Functional Independence Measure (FIM) (96) | 1 (100%) |
|  |  | Pediatric Evaluation of Disabilities Inventory (PEDI) (97) | 1 (100%) |
| Mobility (n=14) | 13 | Six-minute walk test (6MWT) (90) | 4 (29%) |
|  |  | Bruininks-Oseretsky Test of Motor Proficiency (121) | 3 (21%) |
|  |  | Denver Developmental Screening Test (92) | 1 (7%) |
|  |  | Timed 25-foot walk test (T25FW) (91) | 1 (7%) |
|  |  | *MPS III Disability Scale (investigator-developed) (24) | 1 (7%) |
|  |  | ^#^Childhood Health Assessment Questionnaire (CHAQ) (89) | 1 (7%) |
|  |  | Griffiths Scales (122) | 1 (7%) |
|  |  | *MPS Health Assessment Questionnaire (MPS HAQ) (88) | 1 (7%) |
|  |  | Peabody Development Motor Scales II (PDMS-II) (123) | 1 (7%) |
|  |  | Bayley Scales of Infant and Toddler Development (85) | 1 (7%) |
|  |  | 2-minute walk test (2MWT) (124) | 1 (7%) |
|  |  | 3-minute stair climb test (3MSCT) (125) | 1 (7%) |
|  |  | Patient-Reported Outcome Measurement Information System (PROMIS) pain measures (126) | 1 (7%) |
|  |  | General, unclear, or not specified | 9 (64%) |
| Endurance/exercise capacity (n=14) | 7 | Six-minute walk test (6MWT) (90) | 14 (100%) |
|  |  | 3-minute stair climb test (3MSCT) (125) | 6 (43%) |
|  |  | Timed 25-foot walk test (T25FW) (91) | 3 (21%) |
|  |  | 2-minute walk test (2MWT) (124) | 1 (7%) |
|  |  | Expanded timed get-up and go test (127) | 1 (7%) |
|  |  | Cardiopulmonary exercise test (CPET) (128) | 1 (7%) |
|  |  | Modified Balke Treadmill Test (129) | 1 (7%) |
| ***CORE AREA: GROWTH AND DEVELOPMENT*** | | | |
| Cognitive function and early development (n=29) | 16 | Bayley Scales of Infant and Toddler Development (85) | 17 (59%) |
|  |  | ^#^Kaufman Assessment Battery for Children (86) | 13 (45%) |
|  |  | ^#^Vineland Adaptive Behavior Scales^d^ (75) | 7 (24%) |
|  |  | Mullen Scales of Early Learning (MSEL) (87) | 3 (10%) |
|  |  | The Kyoto Scale of Psychological Development (KSPD) (130) | 2 (7%) |
|  |  | Psychoeducational Profile (131) | 2 (7%) |
|  |  | Brunet-Lezine Revised Test (93) | 2 (7%) |
|  |  | Differential Ability Scales (DAS) (132) | 1 (3%) |
|  |  | ^#^Wechsler Intelligence Scale (age-appropriate version) (133) | 1 (3%) |
|  |  | Borel Maisonny Petit (BMP) test (134) | 1 (3%) |
|  |  | Batterie d’Evaluation Cognitive et Sociale (BECS) (Cognitive Skills Assessment Battery) (135) | 1 (3%) |
|  |  | Infant Toddler Quality of Life Questionnaire (IT-Qol) (102) | 1 (3%) |
|  |  | Children's Sleep Habits Questionnaire (CSHQ) (104) | 1 (3%) |
|  |  | Denver Developmental Screening Test (92) | 1 (3%) |
|  |  | Hopkins verbal learning test (136) | 1 (3%) |
|  |  | Rey Auditory Verbal Learning Test (137) | 1 (3%) |
|  |  | General, unclear, or not specified | 10 (34%) |
| Speech abilities (n=5) | 1 | *MPS III Disability Scale (investigator-developed) (24) | 1 (20%) |
|  |  | General, unclear, or not specified | 4 (80%) |
| Fine-motor ability (n=5) | 10 | Bruininks-Oseretsky Test of Motor Proficiency (121) | 3 (60%) |
|  |  | ^#^Functional Dexterity Test (95) | 3 (60%) |
|  |  | Nine-hole peg test (138) | 2 (40%) |
|  |  | Denver Developmental Screening Test (92) | 1 (20%) |
|  |  | Griffiths Scales (122) | 1 (20%) |
|  |  | Bayley Scales of Infant and Toddler Development (85) | 1 (20%) |
|  |  | Peabody Development Motor Scales II (PDMS-II) (123) | 1 (20%) |
|  |  | Purdue Pegboard Test (139) | 1 (20%) |
|  |  | Beery Buktenica Development Test of Visual Motor Integration (140) | 1 (20%) |
|  |  | Rey-Osterrieth Complex Figure (141) | 1 (20%) |
|  |  | General, unclear, or not specified | 2 (40%) |
| ***CORE AREA: PATHOPHYSIOLOGICAL MANIFESTATIONS*** | | | |
| Overall health (clinician assessment) (n=24) | 1 | Clinical Global Impression (CGI) (142) | 1 (4%) |
|  |  | General, unclear, or not specified | 23 (96%) |
| Joint function/range of motion (general) (n=10) | 1 | Six-minute walk test (6MWT) (90) | 1 (10%) |
|  |  | General, unclear, or not specified | 10 (100%) |
| Arthropathy (n=2) | 1 | Six-minute walk test (6MWT) (90) | 1 (50%) |
|  |  | General, unclear, or not specified | 2 (100%) |
| Cardiac function (n=32) | 1 | Modified Balke Treadmill Test (129) | 1 (3%) |
|  |  | General, unclear, or not specified | 32 (100%) |
| Ear, nose, and throat manifestations (n=5) | 1 | *MPS III Disability Scale (investigator-developed) (24) | 1 (20%) |
|  |  | General, unclear, or not specified | 4 (80%) |
| Gastrointestinal manifestations (n=3) | 1 | Pediatric Quality of Life Inventory (PedsQL) (Gastrointestinal Symptoms Module) (143) | 1 (33%) |
|  |  | General, unclear, or not specified | 2 (67%) |
| Lung function (n=16) | 3 | Six-minute walk test (6MWT) (90) | 2 (13%) |
|  |  | Cardiopulmonary exercise test (CPET) (128) | 1 (6%) |
|  |  | Modified Balke Treadmill Test (129) | 1 (6%) |
|  |  | General, unclear, or not specified | 16 (100%) |
| Seizures (including due to epilepsy) (n=2) | 1 | *MPS III Disability Scale (investigator-developed) (24) | 1 (50%) |
|  |  | General, unclear, or not specified | 1 (50%) |
| Spinal manifestations (n=11) | 1 | Six-minute walk test (6MWT) (90) | 2 (18%) |
|  |  | General, unclear, or not specified | 11 (100%) |
| Vital signs (n=23) | 3 | Six-minute walk test (6MWT) (90) | 1 (4%) |
|  |  | Cardiopulmonary exercise test (CPET) (128) | 1 (4%) |
|  |  | Modified Balke Treadmill Test (129) | 1 (4%) |
|  |  | General, unclear, or not specified | 21 (91%) |

^*^MPS-specific tool

^#^Reported by at least one study as being a validated tool.

^a^ In some cases, authors provided partial information about an instrument/tool, but we classified it as “general, unclear, or not specified” as we did not have enough information for proper classification.

^b^ Not mutually exclusive (some studies used or recommended multiple tools to measure the same outcome).

^c^ Some outcomes (e.g., grip strength, mobility) were broadly categorized under activities of daily living (ADL) as they were included as domains in a validated tool used to measure ADL.

Note: different versions of outcome measurement instrument tools within the same family of tools (e.g., PedsQL) were not counted separately

^d^ The Vineland Adaptive Behavior Scales are captured under cognitive function and early development based on the language of outcomes described in the included studies (e.g., cognitive manifestations, neuropsychological function, neurodevelopmental testing, etc.)

**References**

1. Akyol MU, Alden TD, Amartino H, Ashworth J, Belani K, Berger KI, et al. Recommendations for the management of MPS IVA: systematic evidence- and consensus-based guidance. Orphanet J Rare Dis. 2019 Jun 13;14(1).

2. Akyol MU, Alden TD, Amartino H, Ashworth J, Belani K, Berger KI, et al. Recommendations for the management of MPS VI: Systematic evidence- and consensus-based guidance. Orphanet J Rare Dis. 2019 May 29;14(1).

3. Charrow J, Alden TD, Breathnach CAR, Frawley GP, Hendriksz CJ, Link B, et al. Diagnostic evaluation, monitoring, and perioperative management of spinal cord compression in patients with Morquio syndrome. Vol. 114, Molecular Genetics and Metabolism. Academic Press Inc.; 2015. p. 11–8.

4. Fahnehjelm KT, Ashworth JL, Pitz S, Olsson M, Törnquist AL, Lindahl P, et al. Clinical guidelines for diagnosing and managing ocular manifestations in children with mucopolysaccharidosis. Vol. 90, Acta Ophthalmologica. 2012. p. 595–602.

5. Ghosh A, Shapiro E, Rust S, Delaney K, Parker S, Shaywitz AJ, et al. Recommendations on clinical trial design for treatment of Mucopolysaccharidosis Type III. In: Orphanet Journal of Rare Diseases. BioMed Central Ltd.; 2017.

6. Giugliani R, Luz M, Villarreal S, Arellano Valdez CA, Hawilou AM, Guelbert N, et al. Guidelines for diagnosis and treatment of Hunter Syndrome for clinicians in Latin America [Internet]. Vol. 37, Genetics and Molecular Biology. 2014. Available from: www.sbg.org.br

7. González-Gutiérrez-Solana L, Guillén-Navarro E, Del Toro M, Dalmau J, González-Meneses A, Couce ML. Diagnosis and follow-up of patients with Hunter syndrome in Spain: A Delphi consensus. Medicine (United States). 2018 Jul 1;97(29).

8. Hendriksz CJ, Berger KI, Giugliani R, Harmatz P, Kampmann C, Mackenzie WG, et al. International guidelines for the management and treatment of Morquio a syndrome. Vol. 167, American Journal of Medical Genetics, Part A. Wiley-Liss Inc.; 2015. p. 11–25.

9. Kuiper GA, Langereis EJ, Breyer S, Carbone M, Castelein RM, Eastwood DM, et al. Treatment of thoracolumbar kyphosis in patients with mucopolysaccharidosis type I: Results of an international consensus procedure. Orphanet J Rare Dis. 2019 Jan 18;14(1).

10. Langereis EJ, Borgo A, Crushell E, Harmatz PR, Van Hasselt PM, Jones SA, et al. Treatment of hip dysplasia in patients with mucopolysaccharidosis type i after hematopoietic stem cell transplantation: Results of an international consensus procedure. Orphanet J Rare Dis. 2013;8(1).

11. McBride KL, Berry SA, Braverman N. Treatment of mucopolysaccharidosis type II (Hunter syndrome): a Delphi derived practice resource of the American College of Medical Genetics and Genomics (ACMG). Genetics in Medicine. 2020;22(11).

12. Muenzer J, Bodamer O, Burton B, Clarke L, Frenking GS, Giugliani R, et al. The role of enzyme replacement therapy in severe Hunter syndrome-an expert panel consensus. Eur J Pediatr. 2012 Jan;171(1):181–8.

13. Politei JM, Gordillo-González G, Guelbert NB, de Souza CFM, Lourenço CM, Solano ML, et al. Recommendations for Evaluation and Management of Pain in Patients With Mucopolysaccharidosis in Latin America. J Pain Symptom Manage. 2018 Jul 1;56(1):146–52.

14. Scarpa M, Almássy Z, Beck M, Bodamer O, Bruce IA, De Meirleir L, et al. Mucopolysaccharidosis type II: European recommendations for the diagnosis and multidisciplinary management of a rare disease. Vol. 6, Orphanet Journal of Rare Diseases. 2011.

15. Solanki GA, Alden TD, Burton BK, Giugliani R, Horovitz DDG, Jones SA, et al. A multinational, multidisciplinary consensus for the diagnosis and management of spinal cord compression among patients with mucopolysaccharidosis VI. Vol. 107, Molecular Genetics and Metabolism. 2012. p. 15–24.

16. Solano ML, Fainboim A, Politei J, Porras-Hurtado GL, Martins AM, Souza CFM, et al. Enzyme replacement therapy interruption in patients with Mucopolysaccharidoses: Recommendations for distinct scenarios in Latin America. Mol Genet Metab Rep. 2020 Jun 1;23.

17. van der Lee JH, Morton J, Adams HR, Clarke L, Eisengart JB, Escolar ML, et al. Therapy development for the mucopolysaccharidoses: Updated consensus recommendations for neuropsychological endpoints. Mol Genet Metab. 2020 Sep 1;131(1–2):181–96.

18. Wang RY, Bodamer OA, Watson MS, Wilcox WR. Lysosomal storage diseases: Diagnostic confirmation and management of presymptomatic individuals. Genetics in Medicine. 2011;13(5):457–84.

19. Berger KI, Burton BK, Lewis GD, Tarnopolsky M, Harmatz PR, Mitchell JJ, et al. Cardiopulmonary exercise testing reflects improved exercise capacity in response to treatment in morquio a patients: Results of a 52-week pilot study of two different doses of elosulfase alfa. In: JIMD Reports. Springer; 2018. p. 9–17.

20. Burton BK, Berger KI, Lewis GD, Tarnopolsky M, Treadwell M, Mitchell JJ, et al. Safety and physiological effects of two different doses of elosulfase alfa in patients with morquio a syndrome: A randomized, double-blind, pilot study. Am J Med Genet A. 2015 Oct 1;167(10):2272–81.

21. Treadwell M, Harmatz PR, Burton BK, Mitchell JJ, Muschol N, Jones SA, et al. Impact of Elosulfase Alfa on Pain in Patients with Morquio A Syndrome over 52 Weeks: MOR-008: A Randomized, Double-Blind, Pilot Study. J Inborn Errors Metab Screen. 2017 Jul 21;5.

22. Braunlin E, Rosenfeld H, Kampmann C, Johnson J, Beck M, Giugliani R, et al. Enzyme replacement therapy for mucopolysaccharidosis VI: Long-term cardiac effects of galsulfase (Naglazyme®) therapy. J Inherit Metab Dis. 2013 Mar;36(2):385–94.

23. But W, Wong M, Chow J, Chan W, Ko W, Wu S, et al. Enzyme replacement therapy for mucopolysaccharidosis VI (Maroteaux-Lamy syndrome): experience in Hong Kong [Internet]. 2011. Available from: www.hkmj.org

24. Delgadillo V, Del Mar O’Callaghan M, Artuch R, Montero R, Pineda M. Genistein supplementation in patients affected by Sanfilippo disease. J Inherit Metab Dis. 2011 Oct;34(5):1039–44.

25. Garcia P, Phillips D, Johnson JA, Martin K, Randolph LM, Rosenfeld H, et al. Long-term outcomes of patients with mucopolysaccharidosis VI treated with galsulfase enzyme replacement therapy since infancy. Mol Genet Metab. 2021 May 1;133(1):100–8.

26. Harmatz PR, Garcia P, Guffon N, Randolph LM, Shediac R, Braunlin E, et al. Galsulfase (Naglazyme®) therapy in infants with mucopolysaccharidosis VI. J Inherit Metab Dis. 2014 Mar 1;37(2):277–87.

27. Giugliani R, Vieira TA, Carvalho CG, Muñoz-Rojas MV, Semyachkina AN, Voinova VY, et al. Immune tolerance induction for laronidase treatment in mucopolysaccharidosis I. Mol Genet Metab Rep. 2017 Mar 1;10:61–6.

28. Giugliani R, Giugliani L, De Oliveira Poswar F, Donis KC, Corte AD, Schmidt M, et al. Neurocognitive and somatic stabilization in pediatric patients with severe Mucopolysaccharidosis Type i after 52 weeks of intravenous brain-penetrating insulin receptor antibody-iduronidase fusion protein (valanafusp alpha): An open label phase 1-2 trial. Orphanet J Rare Dis. 2018 Jul 5;13(1).

29. Giugliani R, Martins AM, So S, Yamamoto T, Yamaoka M, Ikeda T, et al. Iduronate-2-sulfatase fused with anti-hTfR antibody, pabinafusp alfa, for MPS-II: A phase 2 trial in Brazil. Molecular Therapy. 2021 Jul 7;29(7):2378–86.

30. Giugliani R, Hwu WL, Tylki-Szymanska A, Whiteman DAH, Pano A. A multicenter, open-label study evaluating safety and clinical outcomes in children (1.4-7.5 years) with Hunter syndrome receiving idursulfase enzyme replacement therapy. Genetics in Medicine. 2014;16(6):435–41.

31. Guffon N, Bin-Dorel S, Decullier E, Paillet C, Guitton J, Fouilhoux A. Evaluation of miglustat treatment in patients with type III mucopolysaccharidosis: A randomized, double-blind, placebo-controlled study. Journal of Pediatrics. 2011;159(5):838-844.e1.

32. Haller C, Song W, Cimms T, Chen CY, Whitley CB, Wang RY, et al. Individual heat map assessments demonstrate vestronidase alfa treatment response in a highly heterogeneous mucopolysaccharidosis VII study population. JIMD Rep. 2019 Jun 1;49(1):53–62.

33. Wang RY, da Silva Franco JF, López-Valdez J, Martins E, Sutton VR, Whitley CB, et al. The long-term safety and efficacy of vestronidase alfa, rhGUS enzyme replacement therapy, in subjects with mucopolysaccharidosis VII. Mol Genet Metab. 2020 Mar 1;129(3):219–27.

34. Harmatz PR, Mengel E, Geberhiwot T, Muschol N, Hendriksz CJ, Burton BK, et al. Impact of elosulfase alfa in patients with morquio A syndrome who have limited ambulation: An open-label, phase 2 study. Am J Med Genet A. 2017 Feb 1;173(2):375–83.

35. Hendriksz CJ, Burton B, Fleming TR, Harmatz P, Hughes D, Jones SA, et al. Efficacy and safety of enzyme replacement therapy with BMN 110 (elosulfase alfa) for Morquio A syndrome (mucopolysaccharidosis IVA): a phase 3 randomised placebo-controlled study. J Inherit Metab Dis. 2014 Oct 23;37(6):979–90.

36. Hendriksz CJ, Giugliani R, Harmatz P, Mengel E, Guffon N, Valayannopoulos V, et al. Multi-domain impact of elosufase alfa in Morquio A syndrome in the pivotal phase III trial. Mol Genet Metab. 2015 Feb 1;114(2):178–85.

37. Hendriksz CJ, Berger KI, Parini R, AlSayed MD, Raiman J, Giugliani R, et al. Impact of long-term elosulfase alfa treatment on respiratory function in patients with Morquio A syndrome. J Inherit Metab Dis. 2016 Nov 1;39(6):839–47.

38. Jones SA, Bialer M, Parini R, Martin K, Wang H, Yang K, et al. Safety and clinical activity of elosulfase alfa in pediatric patients with Morquio A syndrome (mucopolysaccharidosis IVA) less than 5 y. Pediatr Res. 2015 Dec 1;78(6):717–22.

39. Jones SA, Breen C, Heap F, Rust S, de Ruijter J, Tump E, et al. A phase 1/2 study of intrathecal heparan-N-sulfatase in patients with mucopolysaccharidosis IIIA. Mol Genet Metab. 2016 Jul 1;118(3):198–205.

40. Kim J, Park MR, Kim DS, Lee JO, Maeng SH, Cho SY, et al. IgE-mediated anaphylaxis and allergic reactions to idursulfase in patients with Hunter syndrome. Allergy: European Journal of Allergy and Clinical Immunology. 2013 Jun;68(6):796–802.

41. Kim KH, Dodsworth C, Paras A, Burton BK. High dose genistein aglycone therapy is safe in patients with mucopolysaccharidoses involving the central nervous system. Mol Genet Metab. 2013 Aug;109(4):382–5.

42. Lund TC, Miller WP, Liao AY, Tolar J, Shanley R, Pasquali M, et al. Post-transplant laronidase augmentation for children with Hurler syndrome: biochemical outcomes. Sci Rep. 2019 Dec 1;9(1).

43. Polgreen LE, Lund TC, Braunlin E, Tolar J, Miller BS, Fung E, et al. Clinical trial of laronidase in Hurler syndrome after hematopoietic cell transplantation. Pediatr Res. 2020 Jan 1;87(1):104–11.

44. Marucha J, Tylki-Szymańska A, Jakóbkiewicz-Banecka J, Piotrowska E, Kloska A, Czartoryska B, et al. Improvement in the range of joint motion in seven patients with mucopolysaccharidosis type II during experimental gene expression-targeted isoflavone therapy (GET IT). Am J Med Genet A. 2011 Sep;155(9):2257–62.

45. Muenzer J, Hendriksz CJ, Fan Z, Vijayaraghavan S, Perry V, Santra S, et al. A phase I/II study of intrathecal idursulfase-IT in children with severe mucopolysaccharidosis II. Genetics in Medicine. 2016 Jan 1;18(1):73–81.

46. EUCTR. https://trialsearch.who.int/Trial2.aspx?TrialID=EUCTR2011-000212-25-GB. 2020 [cited 2023 Mar 6]. An extension to the safety, tolerability and preliminary efficacy study of Idursulfase-IT in patients with Hunter syndrome associated with learning disability. Available from: https://trialsearch.who.int/Trial2.aspx?TrialID=EUCTR2011-000212-25-GB

47. Muenzer J, Beck M, Eng CM, Giugliani R, Harmatz P, Martin R, et al. Long-term, open-labeled extension study of idursulfase in the treatment of Hunter syndrome. Genetics in Medicine. 2011;13(2):95–101.

48. Okuyama T, Eto Y, Sakai N, Nakamura K, Yamamoto T, Yamaoka M, et al. A Phase 2/3 Trial of Pabinafusp Alfa, IDS Fused with Anti-Human Transferrin Receptor Antibody, Targeting Neurodegeneration in MPS-II. Molecular Therapy. 2021 Feb 3;29(2):671–9.

49. Polgreen LE, Kunin-Batson A, Rudser K, Vehe RK, Utz JJ, Whitley CB, et al. Pilot study of the safety and effect of adalimumab on pain, physical function, and musculoskeletal disease in mucopolysaccharidosis types I and II. Mol Genet Metab Rep. 2017 Mar 1;10:75–80.

50. Seo JH, Kosuga M, Hamazaki T, Shintaku H, Okuyama T. Impact of intracerebroventricular enzyme replacement therapy in patients with neuronopathic mucopolysaccharidosis type II. Mol Ther Methods Clin Dev. 2021 Jun 11;21:67–75.

51. Sohn YB, Cho SY, Lee J, Kwun Y, Huh R, Jin DK. Safety and efficacy of enzyme replacement therapy with idursulfase beta in children aged younger than 6years with Hunter syndrome. Mol Genet Metab. 2015 Feb 1;114(2):156–60.

52. Tardieu M, Zérah M, Husson B, De Bournonville S, Deiva K, Adamsbaum C, et al. Intracerebral administration of adeno-associated viral vector serotype rh.10 carrying human SGSH and SUMF1 cdnas in children with mucopolysaccharidosis type IIIA disease: Results of a phase I/II trial. Hum Gene Ther. 2014 Jun 1;25(6):506–16.

53. Tardieu M, Zérah M, Gougeon ML, Ausseil J, de Bournonville S, Husson B, et al. Intracerebral gene therapy in children with mucopolysaccharidosis type IIIB syndrome: an uncontrolled phase 1/2 clinical trial. Lancet Neurol. 2017 Sep 1;16(9):712–20.

54. Whitley CB, Vijay S, Yao B, Pineda M, Parker GJM, Rojas-Caro S, et al. Final results of the phase 1/2, open-label clinical study of intravenous recombinant human N-acetyl-α-D-glucosaminidase (SBC-103) in children with mucopolysaccharidosis IIIB. Mol Genet Metab. 2019 Feb 1;126(2):131–8.

55. Wijburg FA, Whitley CB, Muenzer J, Gasperini S, del Toro M, Muschol N, et al. Intrathecal heparan-N-sulfatase in patients with Sanfilippo syndrome type A: A phase IIb randomized trial. Mol Genet Metab. 2019 Feb 1;126(2):121–30.

56. EUCTR. https://trialsearch.who.int/Trial2.aspx?TrialID=EUCTR2014-003960-20-GB. 2019 [cited 2023 Mar 6]. An Extension Study to Evaluate the Safety and Efficacy of HGT-1410 Administration in Pediatric Patients with Sanfilippo Syndrome Type A. Available from: https://trialsearch.who.int/Trial2.aspx?TrialID=EUCTR2014-003960-20-GB

57. EUCTR. https://trialsearch.who.int/Trial2.aspx?TrialID=EUCTR2013-001479-18-GB. 2019 [cited 2023 Mar 6]. High Dose Genistein in Sanfilippo Syndrome. Available from: https://trialsearch.who.int/Trial2.aspx?TrialID=EUCTR2013-001479-18-GB

58. ClinicalTrials.gov. https://clinicaltrials.gov/ct2/show/NCT03488394. 2021 [cited 2023 Mar 6]. Gene Therapy With Modified Autologous Hematopoietic Stem Cells for the Treatment of Patients With Mucopolysaccharidosis Type I, Hurler Variant. Available from: https://clinicaltrials.gov/ct2/show/NCT03488394

59. ClinicalTrials.gov. https://ClinicalTrials.gov/show/NCT04284254. 2022 [cited 2023 Mar 6]. MT2018-18: Sleeping Beauty Transposon-Engineered Plasmablasts for Hurler Syndrome Post Allo HSCT. Available from: https://ClinicalTrials.gov/show/NCT04284254

60. ClinicalTrials.gov. https://clinicaltrials.gov/show/NCT02055118. 2021 [cited 2023 Mar 6]. Study of Intrathecal Idursulfase-IT Administered in Conjunction With Elaprase® in Pediatric Patients With Hunter Syndrome and Early Cognitive Impairment (AIM-IT). Available from: https://clinicaltrials.gov/show/NCT02055118

61. ClinicalTrials.gov. https://clinicaltrials.gov/ct2/show/NCT02412787. 2022 [cited 2023 Mar 6]. Study of Long Term Safety and Clinical Outcomes of Idursulfase IT and Elaprase Treatment in Pediatric Participants Who Have Completed Study HGT-HIT-094. Available from: https://clinicaltrials.gov/ct2/show/NCT02412787

62. ClinicalTrials.gov. https://clinicaltrials.gov/ct2/show/NCT02455622. 2022 [cited 2023 Mar 6]. Long-term Evaluation on Height and Weight in Patients With MPS II Who Started Treatment at < 6 Years of Age. Available from: https://clinicaltrials.gov/ct2/show/NCT02455622

63. ClinicalTrials.gov. https://ClinicalTrials.gov/show/NCT03566043. 2022 [cited 2023 Mar 6]. RGX-121 Gene Therapy in Patients With MPS II (Hunter Syndrome). Available from: https://ClinicalTrials.gov/show/NCT03566043

64. ClinicalTrials.gov. https://clinicaltrials.gov/ct2/show/NCT04251026. 2022 [cited 2023 Mar 6]. A Study of DNL310 in Pediatric Participants With Hunter Syndrome. Available from: https://clinicaltrials.gov/ct2/show/NCT04251026

65. ClinicalTrials.gov. https://clinicaltrials.gov/ct2/show/NCT04571970. 2023 [cited 2023 Mar 6]. RGX-121 Gene Therapy in Children 5 Years of Age and Over With MPS II (Hunter Syndrome). Available from: https://clinicaltrials.gov/ct2/show/NCT04571970

66. ClinicalTrials.gov. https://clinicaltrials.gov/show/NCT04573023. 2022 [cited 2023 Mar 6]. A Phase Ⅲ Study of JR-141 in Patients With Mucopolysaccharidosis II. Available from: https://clinicaltrials.gov/show/NCT04573023

67. ClinicalTrials.gov. https://ClinicalTrials.gov/show/NCT03423186. 2021. A Study to Assess the Safety and Tolerability of SOBI003 in Pediatric MPS IIIA Patients.

68. ClinicalTrials.gov. https://ClinicalTrials.gov/show/NCT03811028. 2022 [cited 2023 Mar 6]. A Study to Assess the Safety, Tolerability, and Efficacy of Long-term SOBI003 Treatment in Pediatric MPS IIIA Patients. Available from: https://ClinicalTrials.gov/show/NCT03811028

69. ClinicalTrials.gov. https://clinicaltrials.gov/ct2/show/NCT04088734. 2023 [cited 2023 Mar 6]. Gene Transfer Study of ABO-102 in Patients With Middle and Advanced Phases of MPS IIIA Disease. Available from: https://clinicaltrials.gov/ct2/show/NCT04088734

70. ClinicalTrials.gov. https://clinicaltrials.gov/ct2/show/NCT04201405. 2022 [cited 2023 Mar 6]. Gene Therapy With Modified Autologous Hematopoietic Stem Cells for Patients With Mucopolysaccharidosis Type IIIA. Available from: https://clinicaltrials.gov/ct2/show/NCT04201405

71. ClinicalTrials.gov. https://clinicaltrials.gov/ct2/show/NCT02754076. 2020 [cited 2023 Mar 6]. A Treatment Study of Mucopolysaccharidosis Type IIIB (MPS IIIB). Available from: https://clinicaltrials.gov/ct2/show/NCT02754076

72. ClinicalTrials.gov. https://clinicaltrials.gov/ct2/show/NCT03784287. 2023 [cited 2023 Mar 6]. A Treatment Extension Study of Mucopolysaccharidosis Type IIIB. Available from: https://clinicaltrials.gov/ct2/show/NCT03784287

73. ClinicalTrials.gov. https://clinicaltrials.gov/ct2/show/NCT02418455. 2019 [cited 2023 Mar 6]. Study of UX003 Recombinant Human Beta-Glucuronidase (rhGUS) Enzyme Replacement Treatment in Mucopolysaccharidosis Type 7, Sly Syndrome (MPS 7) Patients Less Than 5 Years of Age. Available from: https://clinicaltrials.gov/ct2/show/NCT02418455

74. Shapiro EG, Nestrasil I, Ahmed A, Wey A, Rudser KR, Delaney KA, et al. Quantifying behaviors of children with Sanfilippo syndrome: The Sanfilippo Behavior Rating Scale. Mol Genet Metab. 2015;114(4):594–8.

75. Sparrow S, Cicchetti D V, Saulnier CA. Vineland Adaptive Behavior Scales. 3rd ed. Bloomington, MN: Pearson; 2016.

76. Greenberg LM, Kindschi C, Corman C. TOVA: Test of Variables of Attention, Clinical Manual. Los Alamitos, CA: The TOVA Company; 2018.

77. Conners CK. A teacher rating scale for use in drug studies with children. Am J Psychiatry. 1969;126:884–8.

78. Connors CK. Conners Continuous Performance Test 3rd Edition (Conners CPT 3). Bloomington, MN: Pearson; 2014.

79. Goldsmith HH. Studying temperament via construction of the Toddler Behavior Assessment Questionnaire. Child Dev. 1996;67(1):218–35.

80. HealthActCHQ. CHQ: Child Health Questionnaire. Boston, MA; 2018.

81. Eisengart JB, King KE, Shapiro EG, Whitley CB, Muenzer J. The nature and impact of neurobehavioral symptoms in neuronopathic Hunter syndrome. Mol Genet Metab Rep. 2020;22:100549.

82. Achenbach TM. Manual for the Child Behavior Checklist/4-18 and 1991 Profile. Burlington, VT: University of Vermont; 1991.

83. Reynolds CR. Behavior Assessment System for Children, Third Edition (BASC-3). Bloomington, MN: Pearson; 2015.

84. Gioia GA. BRIEF: Behavior Rating Inventory of Executive Function. Lutz, FL: PAR, Inc.; 2013.

85. Bayley N. Bayley Scales of Infant and Toddler Development. 3rd ed. San Antonio, TX: Harcourt Assessment; 2006.

86. Kaufman A, Kaufman N. Kaufman Assessment Battery for Children (K-ABC) administration and scoring manual. Circle Pines, MN: American Guidance Service; 1983.

87. Mullen EM. Mullen Scales of Early Learning (AGS ed.). Circle Pines, MN: American Guidance Service Inc. National Research Council.; 1995.

88. Batson A, Ahmed A, Yund B, Whitley C, Shapiro E. The MPS health assessment questionnaire: preliminary normative and validity data. Mol Genet Metab. 2014;111(2):S63.

89. Singh G, Athreya BH, Fries JF, Goldsmith DP. Measurement of health status in children with juvenile rheumatoid arthritis. Arthritis Rheum. 1994;37(12):1761–9.

90. American Thoracic Society. ATS Statement: Guidelines for the Six-Minute Walk Test. Am J Respir Crit Care Med. 2002;166:111-7.

91. Motl RW, Cohen JA, Benedict R, Phillips G, LaRocca N, Hudson LD, et al. Validity of the timed 25-foot walk as an ambulatory performance outcome measure for multiple sclerosis. Mult Scler. 2017;23(5):704–10.

92. Frankenburg W, Dodds J, Archer P. Denver II Technical Manual. Denver, CO: Denver Developmental Materials; 1990.

93. Odette B, Lezine I. Echelle de développement psychomoteur de la première enfance : [Test psychologique] BLR Brunet-Lézine révisé Manuel d’instructions. Issy-les-Moulineaux(75) : E.A.P.(Etablissements d’Applications Psychotechniques); 1997.

94. Hogan MJ, Stephens K, Smith E, Jalazo ER, Hendriksz CJ, Edwards LJ, et al. Toileting Abilities Survey as a surrogate outcome measure for cognitive function: Findings from neuronopathic mucopolysaccharidosis II patients treated with idursulfase and intrathecal idursulfase. Mol Genet Metab Rep. 2020;25:100669.

95. Aaron DH, Stegink Jansen CW. Development of the Functional Dexterity Test (FDT): construction, validity, reliability, and normative data. J Hand Ther. 2003;16(1):12-21.

96. Gkouma A, Theotokatos G, Geladas N, Mandalidis D, Skordilis E. Validity and reliability evidence of the Functional Independence Measurement (FIM) for individuals with neurological disorders in Greece. J Med – Clin Res & Rev. 2022;6(5):1-11.

97. Berg M, Jahnsen R, Frøslie KF, Hussain A. Reliability of the pediatric evaluation of disability inventory (PEDI). Phys Occup Ther Pediatr. 2004;24(3):61–77.

98. Herdman M, Gudex C, Lloyd A, Janssen M, Kind P, Parkin D, et al. Development and preliminary testing of the new five-level version of EQ-5D (EQ-5D-5L). Qual Life Res. 2011;20(10):1727–36.

99. Varni JW, Seid M, Rode C. The PedsQL™: Measurement Model for the Pediatric Quality of Life Inventory. Medical Care. 1999;37(2):126-39.

100. Wiklund I, Raluy-Callado M, Stull DE, Jangelind Y, Whiteman DAH, Chen WH. The Hunter Syndrome-Functional Outcomes for Clinical Understanding Scale (HS-FOCUS) Questionnaire: Evaluation of measurement properties. Qual Life Res. 2012;22:875–84.

101. Brazier JE, Harper R, B Jones NM, Thomas KJ, Usherwood T, Westlake L. Validating the SF-36 health survey questionnaire: new outcome measure for primary care. BMJ. 1992;305(6846)160-4.

102. Raat H, Landgraf JM, Oostenbrink R, Moll HA, Essink-Bot ML. Reliability and validity of the Infant and Toddler Quality of Life Questionnaire (ITQOL) in a general population and respiratory disease sample. Qual Life Res. 2007;16(3):445–60.

103. Daltroy L, Liang M, Fossel A, Goldberg M. The POSNA pediatric musculoskeletal functional health questionnaire: report on reliability, validity, and sensitivity to change. J Pediatr Orthop. 1998;18(5):561–71.

104. Owens J, Spirito A, McGuinn M. The Children’s Sleep Habits Questionnaire (CSHQ): psychometric properties of a survey instrument for school-aged children. Sleep. 2000;23(8):1043-51.

105. Horsman J, Furlong W, Feeny D, Torrance G. The Health Utilities Index (HUI): concepts, measurement properties and applications. Health Qual Life Outcomes. 2003;1:54.

106. The KIDSCREEN Group Europe. The KIDSCREEN questionnaires: Quality of life questionnaires for children and adolescents. Pabst Science Publishers; 2006.

107. Jacob E, Mack AK, Savedra M, Van Cleve L, Wilkie DJ. Adolescent pediatric pain tool for multidimensional measurement of pain in children and adolescents. Pain Manag Nurs. 2014;15(3):694–706.

108. Hicks CL, Von Baeyer CL, Spafford PA, Van Korlaar I, Goodenough B. The Faces Pain Scale-Revised: toward a common metric in pediatric pain measurement. Pain. 2001;93(2):173-83.

109. Cleeland C. Brief Pain Inventory Short Form (BPI-SF). 1991.

110. Merkel S, Voepel-Lewis T, Shayevitz J, Malviya S. The FLACC: a behavioral scale for scoring postoperative pain in young children. Pediatr Nurs. 1997;23(3):293–7.

111. Farrar JT, Young JPB, Lamoreaux L, Werth JL, Poole RM. Clinical importance of changes in chronic pain intensity measured on an 11-point numerical pain rating scale. Pain. 2001;94(2):149-58.

112. Varni J, Thompson K, Hanson V. The Varni/Thompson Pediatric Pain Questionnaire. I. Chronic musculoskeletal pain in juvenile rheumatoid arthritis. Pain. 1987;28(1):27–8.

113. Varni JW, Sherman SA, Burwinkle TM, Dickinson PE, Dixon P. The PedsQL^TM^ Family Impact Module: preliminary reliability and validity. Health Qual Life Outcomes. 2004;2:55.

114. Abidin RR. Parenting Stress Index, Fourth Edition (PSI-4). Lutz, FL: Psychological Assessment Resources; 2012.

115. Harrington M, Hareendran A, Skalicky A, Wilson H, Clark M, Mikl J. Assessing the impact on caregivers caring for patients with rare pediatric lysosomal storage diseases: development of the Caregiver Impact Questionnaire. J Patient Rep Outcomes. 2019;3(1):44.

116. Zarit SH, Reever KE, Msg M/, Bach-Peterson J. Relatives of the Impaired Elderly: Correlates of Feelings of Burden. Gerontologist. 1980;20(6):649-55.

117. Beck AI, Steer RA, Carbin MC. Psychometric properties of the Beck Depression Inventory: Twenty-five years of evaluation. Clin Psych Rev. 1988;8(1):77-100.

118. Varni JW, Burwinkle TM, Szer IS. The PedsQL Multidimensional Fatigue Scale in pediatric rheumatology: reliability and validity. J Rheumatol. 2004;31(12):2494-500.

119. Bonuck KA, Goodlin-Jones BL, Schechter C, Owens J. Modified Children’s sleep habits questionnaire for behavioral sleep problems: a validation study. Sleep Health. 2017;3(3):136–41.

120. Fraser J, Gason AA, Wraith JE, Delatycki MB. Sleep disturbance in Sanfilippo syndrome: a parental questionnaire study. Arch Dis Child. 2005;90(12):1239–42.

121. Bruininks R, Bruininks B. Bruininks-Oseretsky Test of Motor Proficiency. 2nd ed. Minneaplois, MN: NCS Pearson; 2005.

122. Luiz DM, Foxcroft CD, Stewart R. The construct validity of the Griffiths Scales of Mental Development. Child Care Health Dev. 2001;27(1):73–83.

123. Folio M, Fewell R. Peabody Developmental Motor Scales, Examiner’s Manual, 2nd ed. Austin, TX: Pro-e; 2000.

124. Pin TW, Choi HL. Reliability, validity, and norms of the 2-min walk test in children with and without neuromuscular disorders aged 6–12. Disabil Rehabil. 2018;40(11):1266–72.

125. McDonald A, Steiner R, Kuehl K, Turbeville S. Clinical Utility of Endurance Measures for Evaluation of Treatment in Patients with Mucopolysaccharidosis VI (Maroteaux-Lamy Syndrome). J Pediatr Rehabil Med. 2010;3:119–27.

126. Cella D, Yount S, Rothrock N, Gershon R, Cook K, Reeve B, et al. The Patient-Reported Outcomes Measurement Information System (PROMIS): progress of an NIH roadmap cooperative group during its first two years. Med Care. 2007;45 5 Suppl 1:S3-11.

127. Wall J, Bell C, Campbell S, Davis J. The timed get-up-and-go test revisited: measurement of the component tasks. J Rehabil Res Dev. 2000;37(1):109-13.

128. DeCato TW, Haverkamp H, Hegewald MJ. Cardiopulmonary Exercise Testing (CPET). Am J Respir Crit Care Med. 2020;201:1–2.

129. Rowland T. Pediatric Laboratory Exercise Testing. Champaing, IL: Human Kinetics Europe Ltd; 1993.

130. Ikuzawa M, Iwachidou S, Oogami R. The Guide of Kyoto Scale of Psychological Development 2001 [Japanese]. Kyoto Kokusai Shakaifukushi Center; 2001.

131. Schopler E, Lansing MD, Reichler RJ, Marcus LM. Psycho Educational Profile: Third edition (PEP-3). Pro-Ed Inc. Austin, TX; 2005.

132. Elliott CD, Salerno JD, Dumont R, Willis JO. The Differential Ability Scales—Second Edition. In D. P. Flanagan & E. M. McDonough (Eds.), Contemporary intellectual assessment: Theories, tests, and issues. The Guilford Press.; 2018. p. 360–82.

133. Wechsler D. Wechsler Intelligence Scale for Children. 5th ed. Bloomington, MN: Pearson; 2014.

134. Borel-Maisonny S. Langage oral et écrit T2: épreuves sensorielles et tests de langage, appréciation de l’état du langage. 8 ed. Delachaux et Niestle; 1997.

135. Adrien J. Batterie d’évaluation cognitive et socio- émotionnelle: Pratiques psychologiques et recherches cliniques auprès d’enfants atteints de TED. Paris: De Boeck; 2008.

136. Brandt J, Benedict R. Hopkins Verbal Learning Test - Revised, Professional Manual, Psychological Assessment Resources. Lutz, FL; 1998.

137. Schmidt M. Rey Auditory Verbal Learning Test: A Handbook. Los Angeles, CA: Western Psychological Services; 1996.

138. Poole JL, Burtner PA, Torres TA, McMullen CK, Markham A, Marcum ML, et al. Measuring dexterity in children using the Nine-hole Peg Test. J Hand Ther. 2005;18(3):348–51.

139. Tiffin J, Asher E. The Purdue Pegboard: Norms and studies of reliability and validity. J Appl Psychol. 1948;32:234–47.

140. Beery K, Buktenica N, Beery N. Beery Buktenica Developmental Test of Visual Motor Integration. Bloomington, MN: Pearson; 2010.

141. Knight J, Kaplan E. Rey-Osterrieth Complex Figure Usage: Clinical and Research Applications, Psychological Assessment Resources. Lutz, FL; 2004.

142. Guy W. Clinical Global Impression (CGI). Rockville, MD: U.S. Department of Health, Education, and Welfare; 1976.

143. Varni JW, Bendo CB, Denham J, Shulman RJ, Self MM, Neigut DA, et al. PedsQL gastrointestinal symptoms module: Feasibility, reliability, and validity. J Pediatr Gastroenterol Nutr. 2014;59(3):347–55.
